# A Later Stone Age quartz knapping workshop and fireplace dated to the Early Holocene in Senegal: The Ravin Blanc X site (RBX)

**DOI:** 10.1371/journal.pone.0329824

**Published:** 2025-09-03

**Authors:** Charlotte Pruvost, Eric Huysecom, Aline Garnier, Irka Hajdas, Alexa Höhn, Laurent Lespez, Michel Rasse, Katja Douze, Sylvain Soriano, Valentine Fichet, Ségolène Saulnier-Copard, Matar Ndiaye, Anne Mayor

**Affiliations:** 1 Laboratory Archaeology of Africa & Anthropology (ARCAN), Faculty of Sciences, University of Geneva, Geneva, Switzerland; 2 Laboratory of Physical Geography (LGP), University of Paris-Est Créteil, France; 3 Laboratory of Ion Beam Physics, ETH Zurich, Zurich, Switzerland; 4 Institut für Archäologische Wissenschaften, Archäobotanik Afrikas, Goethe Universität, Frankfurt am Main, Germany; 5 Laboratory Archéorient, Université Lumière – Lyon 2, Lyon, France; 6 UMR 7041 ArScAn/ AnTET, University of Paris Nanterre, France; 7 Fundamental Institute of Black Africa (IFAN), University Cheikh Anta Diop (UCAD), Dakar, Senegal; 8 Global Studies Institute (GSI), University of Geneva, Geneva, Switzerland; Sapienza University of Rome: Universita degli Studi di Roma La Sapienza, ITALY

## Abstract

Well-dated and well-preserved Later Stone Age sites are unfortunately scarce in West Africa. The few known ones exhibit significant typo-technical variability, reflecting diverse socio-cultural behaviors that remain poorly understood. The Ravin Blanc X (RBX) site in eastern Senegal provides new insights into this period. Excavations at one of the sectors of the site (RBX-1) have revealed a well-preserved Early Holocene occupation, featuring a quartz knapping workshop associated with a fireplace. This site is the latest known LSA occupation in the Falémé valley and bridges a critical gap in the region’s prehistoric sequence. The lithic industry at RBX-1 is dominated by a very homogeneous quartz, which was specifically selected for its high-quality knapping properties. Two main categories of sought blanks were produced: broad, thick, and rectilinear blanks, and elongated, thin and narrow blanks with an oblique distal termination forming a natural asymmetric point. The strong investment in blank standardization from the extraction stage significantly reduced the need for subsequent retouching, which was rarely observed in the RBX-1 lithic assemblage. Comparisons with other LSA sites in West Africa suggest that RBX-1 shares technological similarities with the sites of Fatandi V (Falémé valley, Senegal) and Damatoumou 1 (Ounjougou, Mali), possibly indicating a West African Late LSA Sahelo-Sudanian facies. In contrast, sites located in Guineo-Congolian forest contexts exhibit different knapping strategies and typological choices. The discovery of RBX-1 enhances our understanding of the LSA in West Africa by providing a rare, well-dated stratigraphic context (around 9100 calBP/7100 calBCE) which highlights the complexity of regional lithic traditions and raises new hypotheses about cultural transitions during the Pleistocene-Holocene shift.

## Introduction

**Fig 1:**
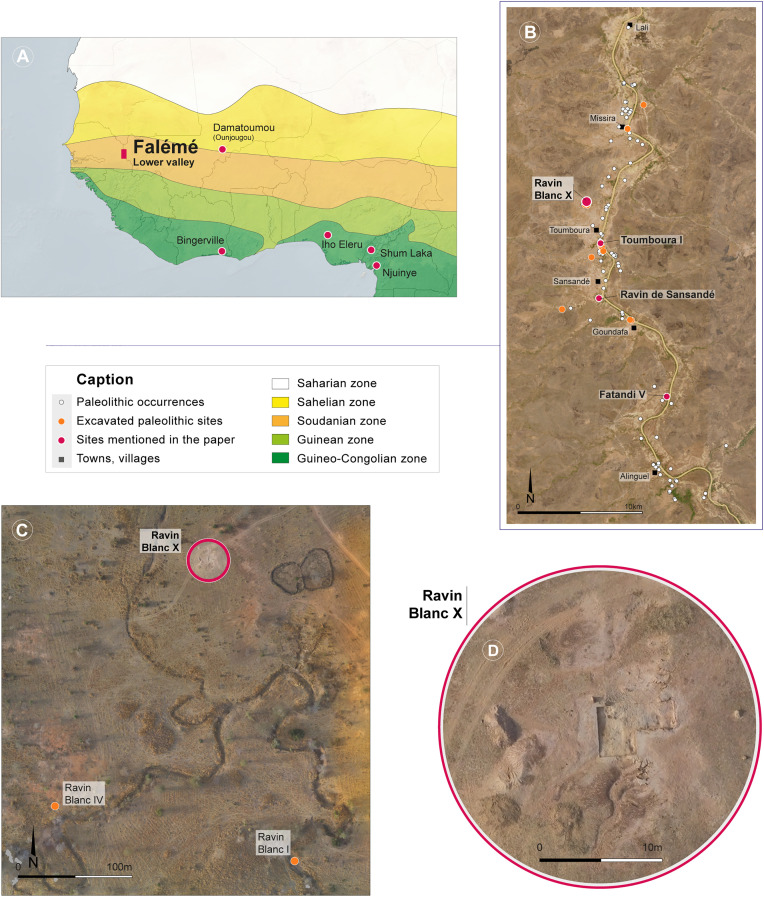
Location of the study area. (A) Climate zones and sites mentioned in the text [1]. (B) Paleolithic occupations identified along the Falémé River. (C) Drone-based orthophotograph of the Ravin Blanc sector. (D) Close-up orthophotograph of the Ravin Blanc X site at the end of excavation (March 2023). Aerial photographs acquired using a Parrot Anafi drone during fieldwork (C and D). Maps and orthophotographs produced using QGIS, MetaShape, and Adobe Illustrator software. Base maps: Natural Earth (A); modified Copernicus Sentinel data [2025] (B). CAD: E. Gutscher and C. Pruvost.

West Africa covers an area of over 6 million km²; unfortunately, stratified and dated Paleolithic and Neolithic sites are scarce and their distribution across the territory is highly uneven. Despite the limited number of sites (Fig 1A), the West African Later Stone Age (LSA) appears diverse. It emerged slightly less than 40,000 years ago in the Guineo-Congolian forests of Cameroon [2–7], at a time when Middle Stone Age (MSA) sites were still present elsewhere in the region [8–10]. The most recent LSA sites in West Africa are dated to the Early Holocene (11,7- ~ 6 ka), and postdate the appearance of the first ceramics in the area, dated from the very beginning of the Holocene [11–13]. In light of this overlap between the LSA and early ceramic traditions, T. Shaw proposed in 1981 the distinction between an “aceramic LSA” and a “ceramic LSA” [14]. Here, however, we have chosen to adopt the terminology most widely used in West African scientific literature, which designates ceramic-bearing sites that predate the advent of metallurgy as “Neolithic” [15]. The sites characterized here as LSA are therefore aceramic.

While the industries from most of the LSA sites of West Africa seem to share a microlithic nature [16], notable typological differences exist between assemblages. The dynamics behind the differences between assemblages are still poorly understood, as are those behind the transitions between the MSA and the LSA, and between the LSA and the Neolithic. Are these different techno-complexes a reflection of coexisting cultural entities with different technical traditions? Do they represent adaptation to different environmental contexts, between savanna and tropical forest, particularly during the Pleistocene-Holocene transition? Do they reflect sites with different functions requiring varied tool sets despite shared knowledge? The complexity of the social, economic, and cultural realities that these transitions represent is difficult to discern, particularly when known sites are so few and often poorly stratified. The high variability in the limited available data indicates transitional processes that are neither linear nor abrupt, and whose boundaries are still poorly defined. In a region as vast and diverse as West Africa, the multiplication of well-documented archaeological discoveries is therefore fundamental to understanding this complex period of cultural, climatic, and environmental transition.

Research conducted over twelve years in the lower Falémé valley (eastern Senegal, Tambacounda region) by the international research program “Human Population and Paleoenvironment in Africa” (HPPA) has revealed a long and significant sequence of occupation, previously partially identified in the 1980s by A. Camara and B. Duboscq [17–20], extending from the Early Stone Age (ESA) to the present day. Until now, three LSA sites had been excavated and dated in the Falémé valley (Fig 1B). These sites are predominantly characterized the use of chert—and to a lesser extent quartz and greywacke—to produce backed pieces (segments or backed blades, depending on the sites) [21,22].

A new LSA site has recently been discovered and excavated on the left bank of the Falémé River: the Ravin Blanc X site (RBX) (Fig 1C and 1D). It takes its name from the nearby Ravin Blanc (French name meaning ‘white ravine’), characterized locally by highly weathered white sedimentary deposits. It is one of many ravines carved during the annual wet season and traversed by intermittent streams flowing into the Falémé. Several archaeological sites have been identified along this ravine, including the ESA and MSA sites of Ravin Blanc I and Ravin Blanc IV [23–27].

In 2017, the discovery of ceramic and lithic material on the surface led to the identification of the RBX site. As no stratified Neolithic site was previously known in this sector of the Falémé valley, initial excavation operations were conducted in 2020 in two sectors: sector 1 (RBX-1) and sector 2 (RBX-2). This article deals more specifically with RBX-1, which delivered the most promising results [28,29]. More information about the excavations and material discovered at RBX-2 is available in the 2020 operation report [27].

RBX-1 has yielded two main occupations: an LSA occupation, dated to the Early Holocene (layer 2) and a final Neolithic occupation dated to the 11th-13th centuries BCE (layer 4). The LSA occupation of layer 2, which postdates the earliest ceramics in West Africa but has not yielded any itself, makes RBX the most recent LSA site known in the Falémé valley, and one of the most recent in West Africa. Benefiting from exceptional preservation conditions in a region where stratified sites are otherwise rare, this occupation is characterized by a fireplace associated with a quartz knapping workshop, both of which could be excavated entirely in situ. Such preservation conditions led us to implement diverse studies (grain size, phytoliths and anthracology), in an interdisciplinary approach, with the aim of attempting to reconstruct the paleoenvironment contemporary with the site’s occupation as precisely as possible. The quartz knapping workshop, at first glance very different from other LSA sites in the Falémé valley that favor the use of chert, has been the subject of an in-depth technological study aimed at identifying the knapping methods employed at the site and the purpose of the knapping activities.

This article presents the history of research conducted at the RBX site, as well as the methods and strategies of excavation and analysis adopted in accordance with the reality of the site. The results of the various novel studies conducted on this site, first paleoenvironmental and then technological analysis of the lithic material, are detailed and placed in a broader regional context, with the aim of contributing to the many questions about the LSA and Early Holocene in West Africa.

## Methods

### Excavation strategy

The excavation of the Ravin Blanc X site was carried out in agreement with the government of the Republic of Senegal (Collaboration agreement between the Institut Fondamental d’Afrique Noire and the ARCAN laboratory of the University of Geneva dated January 12, 2018; Research permit issued by the Direction du Patrimoine Culturel No. 0004 on July 2, 2021) (S1 File).

During the first operation in 2020, RBX-1 was the subject of a 1.50 x 1 m test pit, located where the assemblage of ceramic sherds and lithic material that led to the discovery of the site was exposed (13°59’13.43“N, 12°13’25.72”W, alt. +47m). RBX-2 was established 25 meters northeast of RBX-1 (13°59’13.85”N, 12°13’25.02”W, alt. + 48m), in a higher position on the site, in hopes of finding this industry at depth and thus in situ; 3 m² were excavated there. Two additional excavation campaigns were conducted at RBX-1: 14 m² were excavated during the 2021 fieldwork, and 7 more m² in 2023, for a total of 22.50 m² over the three missions. The squares were labelled with letters on the north-south axis, from D to H, and with numbers on the west-east axis, from 0 to 5.

In 2020, the two sectors were excavated by one-meter squares and by arbitrary horizontal spits of 10 cm thickness [27]. The recorded sections allowed understanding of the site’s stratigraphy, which made possible excavation by sedimentary layer the following year. The material was collected by square and by spit in 2020; by square and by sedimentary layer in 2021, with the altitude of significant pieces being recorded before collection using a builder’s level [28]. Numerous small pieces were also collected during dry sieving, using a 6 mm mesh.

The quartz knapping workshop was subject to a distinct collection protocol. It was excavated by fine spits during which a maximum of lithic pieces was uncovered and documented in place before collection. Each of these spits was photographed, all uncovered artifacts were drawn in plan and their coordinates recorded using a total station. All sediment extracted during these spits was sieved using the finest mesh available at the time, namely a 3 mm mesh.

The last operation carried out in 2023 aimed to extend the excavation around the knapping workshop, which was then at the edge of the excavation area. The 7 m² opened that year were excavated by sedimentary unit, and material from the upper layers (layers 5, 4 and 3) was collected by square and by layer. The material from layer 2 to which the knapping workshop belongs was systematically coordinated using a total station. The discovery during sieving in 2021 of numerous fine fraction elements (< 5–10 mm) led us to manufacture even finer sieves than those employed during the previous mission. The sediment extracted during the excavation of all sedimentary units was thus sieved during this last operation using a 2 mm mesh.

For these three excavation operations, the altitudes were calculated from a reference zero point marked by a nail driven into a tree. The altitudes reached at the end of each spit (arbitrary or sedimentary) were measured at the center of each excavated square meter.

Three sediment samples were collected during the 2021 excavation, for grain size and phytolith analyses: one within the fireplace (sample #1, square E1), one within the workshop (sample #2, squares F1 and G1) and one in layer 1, directly beneath the workshop area (sample #3, squares F1 and G1).

### Grain size analysis

The three samples were first dried overnight in an oven at 45°C. Organic matter was then eliminated with 35% hydrogen peroxide. Particles from 10 nm to 2 mm were then measured by laser granulometry using the Malvern Mastersizer 3000. For this, the samples were previously dispersed using a 5‰ sodium hexametaphosphate solution then sieved at 2 mm. The fraction larger than 2 mm was recovered, dried, and sieved through a column of sieves from 2 to 8 mm. Each fraction retained was weighed and integrated into the results of the fraction smaller than 2 mm. The analysis of this fraction was carried out by laser diffraction of 3–4 aliquots of each sample, each aliquot being measured 3 times for 30 seconds. All data was then averaged and analyzed using the GRADISTAT calculation sheet [30].

### Phytolith extraction and classification

Phytolith extraction was performed from approximately 20g dry sediment samples according to standard protocols [31], which include 4 main steps: 1) Destruction of organic matter with hydrogen peroxide (H_2_O_2_ 33%, at 90°C); 2) Clay dispersion with sodium hexametaphosphate (SHMP – 40 g/l); 3) Separation of sands by sieving at 250 µm and clays by decantation; and 4) Phytolith extraction by heavy liquid separation using diluted sodium polytungstate (SPT) at a density of 2.30–2.35. The phytoliths were then dried and mounted between slide and coverslip with immersion oil, allowing three-dimensional observation and easy manipulation.

Phytolith morphotypes were subsequently identified according to the International Code for Phytolith Nomenclature (ICPN 2.0) [32]. In this study, the so-called “general” approach was used [33]. Its objective is to study all diagnostic and non-diagnostic morphotypes observed and compare their relative frequency between assemblages.

The observed phytoliths could be classified into three main categories: 1) Morphotypes produced by woody dicotyledons, which include sclereids with the cylindrical facetate morphotype as well as spheroid ornate and nodular morphotypes; 2) spheroid echinate specific to the Arecaceae (Palm) family; and 3) Diagnostic morphotypes of Poaceae and herbs. In the latter category, we find notably the grass silica short cells phytoliths (gsscp) which are of particular interest in reconstructing past vegetation communities as the high taxonomic specificity of these morphotypes allows differentiation between Poaceae subfamilies [34–36]. Thus, the bilobate and cross morphotypes are typical of the Panicoideae subfamily, which are tall grasses populating mesophilic savannas. The saddle morphotype is mainly produced by Chloridoideae, low grasses of xerophilic savannas. We also find in this group of phytoliths the bulliform flabellate morphotype, produced in Poaceae and Cyperaceae leaves, as well as the polygonal scrobiculate morphotype, diagnostic of Cyperaceae. Finally, a last group of phytoliths characterized by their shape (elongate, blocky) but not diagnostic of a specific species, family, or group were also counted.

### Anthracological analysis

Three samples of macroscopic wood charcoal were analyzed, all three from layer 2: two samples from inside the fireplace (samples #A and #B), and one from outside of the fireplace (sample #C) (Table 1). The hand-picked charcoal fragments were often still stuck to the clayey sediment. To separate the fragments from the sediment for analysis, they were dissolved in water and poured over a sieve with a 500 µm mesh size. This was not necessary for sample #B, for which most of the charcoal fragments had already been separated from the clayey matrix. For identification, sufficiently large fragments (> 4 mm long) were hand-fractured into the three wood anatomical planes, analyzed with a Leica DM 4000 incident microscope (magnifications 50-500x), and compared with wood anatomical slides from the Goethe University Archaeobotanical Reference Collection.

**Table 1:** Anthracological samples analyzed.

Samples	Layer	Square – Context	Altitude	Analyzable fragments (n = 64)
#A	2	E1 - Fireplace	−187 cm	30
#B	2	F1/G1 – Top of the fireplace	−181 cm	25
#C	2	E1 – Outside of the fireplace	−183 cm	9

### Radiocarbon dating

Prior to radiocarbon analysis samples of charcoal were treated with acid and base to remove contamination with carbonates and humic acids, respectively [37,38]. In the next step, clean and dry sample material were weighed into aluminum boats for a combustion in the elemental analyzer and a subsequent graphitization [39]. The graphite samples pressed into the aluminum cathodes were analyzed using the MICADAS system at the ETH Zurich [40]. Radiocarbon ages were calculated following the convention [41] and calibrated at 2σ using the OxCal online program [42,43] and Intcal20 calibration curve [44]. All radiocarbon dates mentioned in this article, including those from other publications, have been (re)calibrated using the same program and calibration curve to ensure consistency in comparisons.

### Lithic material study

The entire archaeological material discovered during the Ravin Blanc X excavations (collected in situ and from sieving) was inventoried and studied (S1 Table). It was granted temporary exportation permits for the duration of the study (Authorization for the transport of archaeological objects and exit from the national territory No. 00000149 dated April 15, 2021, and No. 00000190 dated March 24, 2023). The study of lithic material found in layer 2 of RBX-1, which is the focus of this article, was conducted using a typo-technological approach. The goal of this method is to propose a reconstruction of the *chaînes opératoires* (or reduction sequences) employed on the site, from raw material procurement, core exploitation and maintenance, the eventual retouch of the blanks, to material and site abandonment [45–52].

The raw materials constituting the corpus are well-documented in the lower Falémé valley, so their identification presented no particular problem [29]. Regarding the quartz, which represents 99% of this assemblage, various characteristics were observed both macroscopically, with the naked eye, and mesoscopically, using a binocular loupe. The aim was to highlight the possible presence of distinct types of quartz within the assemblage. The characteristics retained for study are the following: structure (micro- or macrocrystalline), color and transparency of the crystals (transparent or opaque), break surface (smooth, slightly irregular, irregular), brilliance (matte or shiny) and the overall transparency of the material (translucent, semi-translucent, opaque).

The excavation allowed for the collection of almost all dimensional classes of artifacts, notably through sieving. As mentioned earlier, the resulting assemblage is essentially composed of vein quartz. Vein quartz is a rock composed of quartz crystal agglomerates, within which shock waves preferentially propagate along crystal attachment interfaces. This results in an overall prismatic fracture and an angular product morphology [53–55]. Consequently, quartz knapping activities produce numerous angular waste, often difficult to distinguish from natural debris, as well as numerous small, non-intentional, spontaneous, and/or parasitic elements (small flakes and flake fragments) [53,54,56–61]. While in a knapping workshop context, we can consider that a significant portion of these elements is certainly of anthropogenic origin, these micro-elements are nonetheless non-significant from a technological point of view as they cannot be reintegrated into reduction sequences.

This situation necessitated establishing a dimensional limit to constitute the corpus for detailed study. Rather than setting an arbitrary limit, we considered the dimensions of products whose negatives are observable on the cores and which themselves testify to the recurrence of the debitage they undergo by the similar product scars they bear. As these mostly measure over 15 mm (as do the scars of such products), the dimensional limit of the study was set at 15 mm. Thus, angular waste, natural pieces, and artifacts measuring less than 15 mm were excluded from detailed study.

The descriptive characteristics documented during the piece study are those classically used in technological approaches: i.e., typo-technological category, fragmentation, percentage and location of natural surfaces, organization and direction of previous scars, breaks and accidents, surface condition, description of the proximal zone (platform, bulb, lip), description of retouch [49]. The morphology and general structure of the cores were described: number, organization, and description of exploitation surfaces, opposite surfaces, platform surfaces, and the most recent debitage scars (S1 Table).

All pieces were weighed using an electronic balance precise to 0.1 g. Each piece was measured using a digital caliper: length (technological sense), width, and thickness (at the widest and thickest points of the pieces, excluding the bulb), platform thickness when preserved. Various angles (retouch, Exterior Platform Angle – EPA, Interior Platform Angle – IPA) were measured using a mechanical goniometer. Distal terminations, when preserved, were described in plan (straight, concave, convex, left oblique, right oblique, pointed, complex) and in section (feather, snap, step, hinge, plunging, complex), using morphological terms defined by J. Kamminga et al. and completed by J. Coppe and V. Rots [62,63].

Several categories were distinguished to designate elongated products: blades, bladelets, and elongated flakes. Traditionally, blades are defined as flakes whose length is at least twice their width [64]. Bladelets, in turn, are small blades, with a definition that varies between corpuses [49]: here, we chose to consider as bladelets all blades whose width is less than 10 mm, without presuming they represent distinct production objectives. Elongated flakes include all flakes whose length is between 1.5 to 2 times their width [65]. These dimensional criteria were applied strictly, even in cases of object fragmentation, without presuming their original dimensions. Indeed, speaking of a “blade fragment” for an object that currently has the dimensions of a flake is an interpretation that can prove inaccurate – particularly with quartz, whose fracturing properties result in snap terminations or rebounds, often difficult to distinguish from breaks occurring during the knapping [55]. This choice of characterization may, however, introduce the opposite bias, i.e., considering as flakes products that may not have originally been flakes. Nevertheless, this choice seemed preferable considering the material studied. Indeed, the blanks that seem to have been sought on the site have common, transverse characteristics, sometimes independent of the length of the initial blank (straightness of edges, thickness of blanks, etc.). Therefore, considering a fragment with rectilinear edges and ridges to be a flake fragment seems more consistent with the reduction sequences identified at the site than assuming that all pieces of this type are blade fragments. In any case, these attributions must be considered carefully, particularly when dealing with flake fragments.

On this subject, regularity of edges and ridges are sometimes criteria used to define these elongated products. However, the constraints implied by knapping a material like quartz make true “standardization” of products difficult, such that it seems irrelevant to consider these regularity criteria as necessary for defining blades or elongated flakes. We therefore chose not to integrate these into our definition of elongated products and to document them separately.

Particular attention was paid to refitting throughout the study, with a specific focus on cores. Approximately thirty refittings were carried out, which greatly contributed to understanding the reduction sequences [66–68].

Most material was washed with water, with only very occasional use of an abrasive tool (like a toothbrush) and after verifying that no visible macroscopic residue was present on the pieces (i.e., colorants or adhesives). Refittings were held together for the duration of the study using an adhesive paste, which was removed during final reconditioning of the objects for their return. Pieces broken during excavation were reassembled using a mixture of Paraloid B72 and acetone. This mixture, used in heritage conservation, has the advantages of being colorless, stable, resistant over time, and reversible, as it can be dissolved by applying acetone [69]. A matting spray was applied to some pieces, particularly the most translucent ones, to improve their readability; judging the result inconclusive, this practice was not generalized.

## Results

### Stratigraphic and chronological data

**Fig 2:**
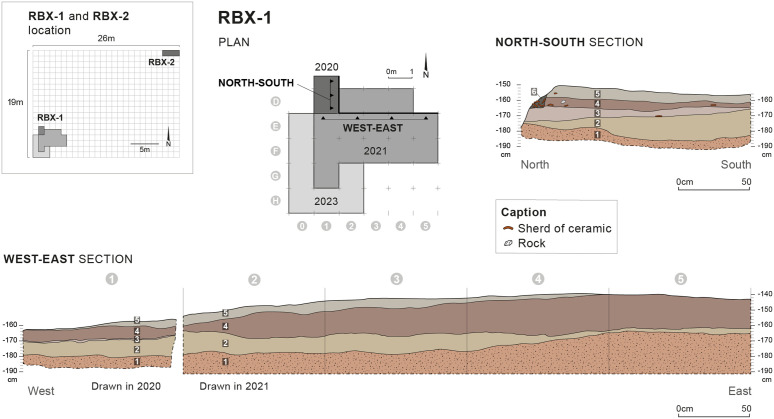
Excavation plan and stratigraphic survey of RBX-1. Drawings: D. Glauser and E. Huysecom. CAD: D. Glauser, E. Gutscher and C. Pruvost.

Six main stratigraphic units were identified at RBX-1, numbered from 1 to 6, with a total stratigraphic thickness of approximately 35–50 cm (Fig 2). For RBX-2, four sedimentary units were identified, numbered from 1 to 4. Pieces of charcoal were discovered in several units from both sectors and allowed radiocarbon dating (Table 2). Some stratigraphic correlations could be made between the two sectors, particularly regarding the recent upper layers. Together, stratigraphic sequences and chronological data from both sectors allowed establishing a phasing of the site’s occupation (Table 3). Nevertheless, the survey carried out at RBX-2 yielded very little material, which is generally poorly informative. The ceramics found were extremely fragmented and no decoration could be identified. As for the lithic material, few artifacts have been identified, and these are mostly non-diagnostic from a typo-technological point of view (unretouched quartz flakes, angular waste...). The few retouched elements discovered consist of a chert segment and two fragments of a polished hematite axe from colluvial levels. Thus, considering the limited input of the RBX-2 survey, only the sedimentary layers of RBX-1 are described here. For more information about the units identified at RBX-2 refer to the 2020 operation reports [27].

**Table 2:** Dates obtained on charcoals from Ravin Blanc X (sectors 1 and 2).

Site	Lab #	Layer	Square	Precise context	Z (cm)	Uncal. age (BP)	Cal. age (calBP)	Cal. age (calBCE/CE)
RBX-1	ETH-113980	4	F2	Base of l. 4/ top of l. 3	−169	2940 ± 24	3173−2998	1224−1049 calBCE
RBX-1	ETH-113981	2	E1	Upper part of the layer	−175	8227 ± 30	9397−9028	7448−7079 calBCE
RBX-1	ETH-113985	2	F1	Fireplace (top)	−182	8162 ± 42	9271−9006	7322−7057 calBCE
RBX-1	ETH-113983	2	E1	Fireplace	−184	8101 ± 30	9127−8988	7178−7039 calBCE
RBX-1	ETH-113984	2	E1	Fireplace (base)	−187	8106 ± 30	9127−8990	7178−7041 calBCE
*RBX-1*	*ETH-113977*	*4*	*F4*	*–*	*−160*	*1239 ± 23*	*1268−1072*	*682-879 calCE*
*RBX-1*	*ETH-113979*	*4*	*F4*	*–*	*−163*	*1230 ± 23*	*1260−1069*	*691-881 calCE*
*RBX-1*	*ETH-113982*	*2*	*E3*	*–*	*−178*	*351 ± 23*	*487−316*	*1464-1635 calCE*
RBX-2	ETH-106227	4	–	Base of the layer	−76	974 ± 22	929−793	1021-1157 calCE
RBX-2	ETH-106228	3	–	Middle part of the layer	−92	1290 ± 22	1284−1176	667-774 calCE
RBX-2	ETH-106230	3	–	Base of the layer	−116	1936 ± 23	1931−1747	20-203 calCE
RBX-2	ETH-106229	2	–	–	−112	2544 ± 23	2745−2513	796−564 calBCE
RBX-2	ETH-106231	2	–	–	−127	2183 ± 23	2309−2114	360−165 calBCE

2 σ calibration performed on OxCal v.4.4.4 [43], using the IntCal20 calibration curve for the northern hemisphere [44]. In grey, dates associated with LSA occupation of layer 2. In italics, dates from disturbed contexts, not retained.

**Table 3:** Correspondence table of layers and dates obtained for the two Ravin Blanc X sectors.

Phases	RBX-1			RBX-2			Chrono-cultural attribution
	Layer	Calibrated age	Number of dates	Layer	Calibrated age	Number of dates	
VI	6	–	0	*Phase absent from RBX-2*			Undetermined, Late Holocene
V	*Phase absent from RBX-1*			4	1021-1157 calCE	1	Medieval period, Late Holocene
IV	*Phase absent from RBX-1*			3	20-774 calCE	2	Iron Age, Late Holocene
III	5	–	0	2	796−165 calBCE	2	Final Neolithic, Late Holocene
	4	1224−1049 calBCE	1				
	3	–	0				
*HIATUS (± 6000 years)*							
**II**	**2**	**9397−8988 calBP** (7448−7039 calBCE)	**4**	*Phase absent from RBX-2*			**LSA, Early Holocene**
I	1	–	0	1	–	0	Sterile, Pleistocene

#### Layer 6.

Layer 6 consists of a highly localized dark gray silty-sandy sediment pocket, containing one of the ceramic clusters identified on the surface. 81 sherds were discovered here. Except for 5 sherds with polished red slip surfaces, poor preservation of recovered sherds prevents the identification of potential surface decorations or original sherd thickness. The majority show temper composed of crushed sandstone, small quartz grains or laterite grains. This unit has not been dated.

#### Layer 5.

Layer 5 consists of light beige-gray silty-sandy colluvium with a very friable character. This layer most likely consists of colluvial deposits that reworked the top of layer 4, as evidenced by the very similar archaeological material found in both layers. Layer 5 yielded 36 small, highly eroded and fragmented sherds, the majority of which show crushed sandstone temper. 149 lithic elements were also identified, of which 82 are quartz, 34 are chert, 29 are greywacke and 4 are hematite. Most of this material consists of unmodified flakes, except for a polished hematite flake, a fragmented *micro-tranchet* roughout (i.e., a microlith with an unretouched oblique sharp edge and two retouched divergent edges, generally through abrupt retouch) in chert and 6 cores in chert (n = 3), quartz (n = 2) and greywacke (n = 1). Only one piece of charcoal was discovered here, but unfortunately it was too small to be dated.

#### Layer 4.

Layer 4 consists of brown-gray silty-sandy colluvial sediments, very fine and extremely compact. It is characteristic of sediment compaction at the top of an eroding glacis. While the upper part of the layer was likely reworked by the deposition of layer 5, the rest of the layer appears intact and has yielded a very rich archaeological assemblage.

95 sherds were discovered in this layer, most too eroded to determine their thickness and any decorations. The only decoration identified on a sherd consists of rolled impressions made using a fine twisted cord. The temper of these sherds is mainly characterized by mixed sand grains, crushed sandstone fragments and laterite nodules.

**Fig 3:**
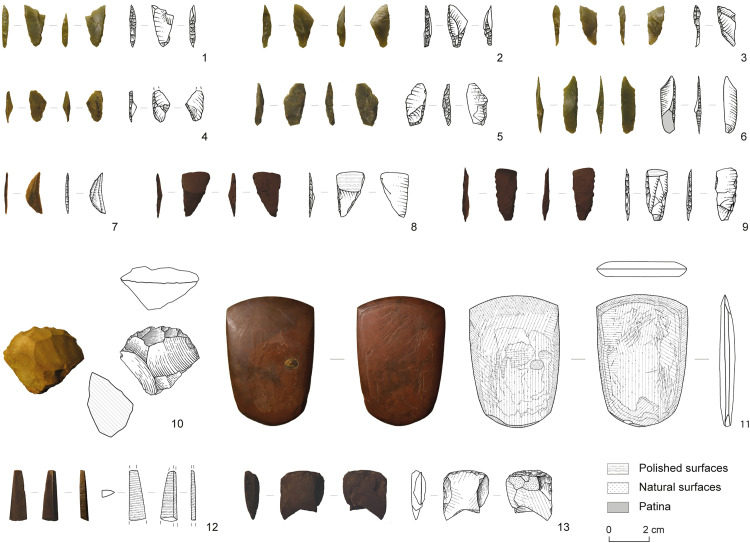
Lithic material found in layer 4 of RBX-1. (1–6) Chert *micro-tranchets*. (7) Chert segment. (8–9) Hematite *micro-tranchets*. (10) Greywacke scraper. (11) Hematite polished axe. (12) Hematite polished needle fragment. (13) Hematite polished and retouched adze fragment. Drawings: E. Gutscher. Photos: C. Pruvost.

The lithic material comprises 381 pieces, of which 159 are quartz, 118 are chert, 89 are greywacke and 15 are hematite. The tool kit from this layer is very rich. It includes polished elements, all in hematite, among which an axe blade (Fig 3, no. 11), a needle or point fragment (Fig 3, no. 12) and a fragmented adze blade showing semi-abrupt retouch (Fig 3, no. 13). The assemblage includes several *micro-tranchets* in chert (Fig 3, no. 1–6) and in hematite (Fig 3, no. 8 and 9), some of which show polished surfaces. A chert segment (Fig 3, no. 7), a greywacke scraper (Fig 3, no. 10), a fragment of a lanceolate point in hematite and various retouched flakes complete this tool set. Some macrolithic elements in greywacke are also notable, namely 2 grindstone fragments, a mortar fragment reused as a grinder and a possible hammer stone. Finally, 5 quartz cores, 2 greywacke cores, one pyramidal core and one core roughout in chert were discovered. The rest of the lithic material from layer 4 consists of unmodified flakes.

Four pieces of charcoal were collected from this layer, one of which, being too small, could not be dated (ETH-113978). The other three provided the following dates: 1224−1049 calBCE (ETH-113980), 682−879 calCE (ETH-113977), and 691−881 calCE (ETH-113979) (Table 2). The first of these three dates comes from charcoal sampled from the heart of the layer, at −169 cm altitude, and suggests a Final Neolithic occupation. Such a date is consistent with the material found in this layer: pottery, polished lithic tools, grinding equipment, etc. *Micro-tranchets* themselves are a type of armature that is rarely documented in West Africa. To our knowledge, the only stratified context in which such objects have been found is the Neolithic site of Fanfannyégéné I in Mali [70]. Some Senegalese oblique-edged microliths defined as “Belairian” by J. Monge also appear similar to the *micro-tranchets* discovered at RBX-1, as do certain armatures identified on the surface during surveys in the Niokolo-Koba National Park, also in Senegal [71,72].

The last two dates (682−879 calCE and 691−881 calCE) come from charcoals sampled from the top of the layer (at −160 and −163 cm altitude respectively). These dates, very recent compared to the previous one, postdate the appearance of iron metallurgy in the Falémé valley and therefore cannot be associated with a Neolithic context [73]. We must therefore consider the possibility that layer 4 consists of several colluvial deposits from the Late Holocene, indistinguishable during excavation, or that these pieces of charcoal are intrusive and result from percolation.

#### Layer 3.

Layer 3 consists of a succession of thin stratified deposits of white to very light beige fine sands. Like layer 4, this layer is also very indurated, which is characteristic of sediment compaction at the top of an eroding glacis. Layer 3 was identified during the first site survey in 2020. Its thickness reduces towards the south profile where it forms a thin white line that disappears gradually. Given that the excavation was extended in subsequent years to the south of the initial test pit, layer 3 was not identified over most of the excavated area, where it was likely absent.

#### Layer 2.

A quartz knapping workshop associated with a fireplace was discovered in layer 2. Lithic material was also discovered outside the knapping workshop, in the rest of the layer, but no ceramic sherds are present from this sedimentary unit onward. This study focuses on this layer, the results of which are detailed later.

Grain size analyses performed on the two sediment samples from layer 2, collected respectively from within the fireplace and within the knapping workshop (Table 4, samples #1 and #2), are very close and confirm they belong to the same sedimentary layer. The main part of the sediment consists of beige-brown coarse silt (around 30%) and fine sand (around 36%). This composition corresponds to wind-blown sediments of the region. The absence of gravel in this layer confirms the low level of disturbance by runoff. The sediment’s structure in small, extremely compact, and hardened aggregates indicates a pedological evolution leading to the formation of a soil level, likely the upper part of the paleosol corresponding to layer 1. Although subtle, the slight difference in clay and silt content between the two samples can perhaps be explained by a more evolved pedogenesis for sample #2 and a higher ash content for sample #1.

**Table 4:** Results of grain size analyses carried out on three RBX-1 samples.

Sample	Layer	Square – Context	Clays	Silts	Sands	Gravels
#1	2	E1 - Fireplace	11,3%	49,2% of which 30,3% coarse silts	39,5% of which 36,6% fine sands	0%
#2	2	F1/G1 – Knapping workshop	12,6%	46,6% of which 29,3% coarse silts	40,8% of which 36,9% fine sands	0%
#3	1	F1/G1 – Under the knapping workshop	10,9%	44,6% of which 24,6% coarse silts	28,2% of which 26,1% fine sands	16,3%

**Fig 4:**
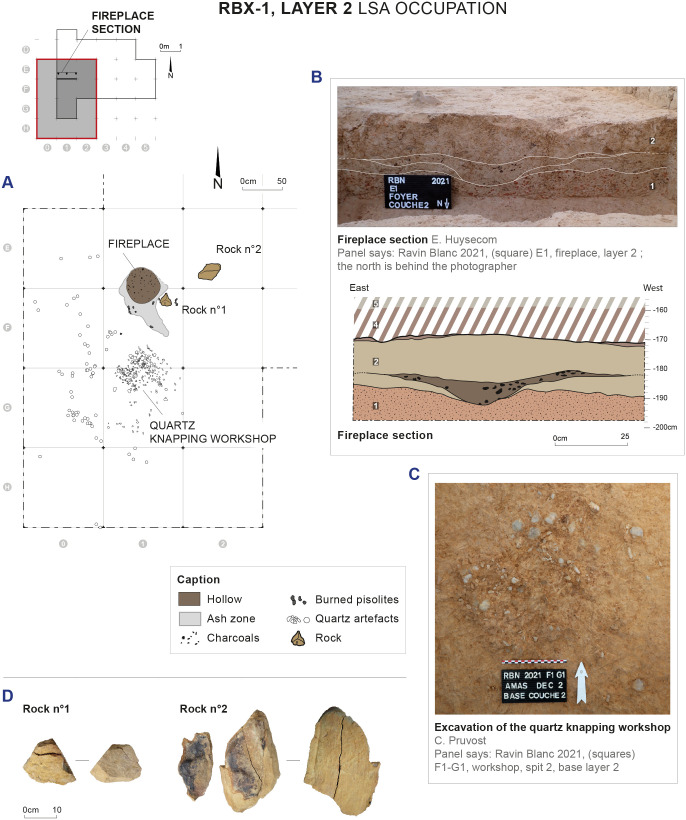
Fireplace and quartz knapping workshop discovered in layer 2 of RBX-1 and dated to the Early Holocene. Drawings and photos: E. Huysecom and C. Pruvost. CAD: D. Glauser, E. Gutscher and C. Pruvost.

The knapping workshop is mainly located in squares F1 and G1, but the highest density of material is contained within a more restricted area of about 80 cm in diameter (Fig 4A and 4C). The workshop consists mainly of quartz micro-elements, such as angular waste and flakes/flake fragments smaller than 15 mm (n = 985/1193). Although providing little information from a typo-technical point of view, the presence of these micro-elements and the extremely localized spatial distribution of the whole knapping workshop are additional evidence supporting the very limited impact of post-depositional disturbance processes.

Located in squares E1 and F1, thus immediately north of the knapping workshop, the fireplace takes the form of a small circular hollow, dug from a level located in the middle of the fill of layer 2 (z = −180/182 cm) and carved into the top of layer 1 (z = −194 cm), for a total depth of 12–14 cm (Fig 4B). The maximum diameter of the hollow is 65 cm (east-west). It is surrounded by a rubefaction zone, characterized by very ashy sediment containing numerous small charcoal pieces and burned pisolites (Fig 4A). Approximately thirty small charcoal pieces (between 2 and 6 mm) were collected in the hollow during excavation and all the sediment contained in the hollow was sampled; numerous charcoal pieces of smaller dimensions are present in this sediment.

Two greywacke blocks were found in the immediate vicinity of the fireplace, though their association with this structure is unclear (Fig 4A and 4D). Neither of the two rocks shows traces of rubefaction on their external surface. They present a neocortex (5–10 mm thick) resulting from intense alteration typically found in lateritic soils. This has separated, through desquamation, from one of the faces of rock no. 2 (Fig 4D). At the interface, the rock presents a reddened or even blackened appearance, which is likely linked to the presence of iron oxides.

Among the charcoal collected in situ, three samples have been dated. Selected from three distinct points in the fireplace (at the top, z = −182 cm; in the center of the fireplace, z = −184 cm; and at the base of the fireplace hollow, z = −187 cm), they have provided remarkably consistent dates, situated between 9271 and 8988 calBP (ETH-113985, ETH-113983, ETH-113984, Table 2). Outside the fireplace, in layer 2, two additional charcoal samples were collected for dating. One of them, coming from a context identified during excavation as probably disturbed, provided an extremely modern date around the 15th and 17th centuries CE (ETH-113982). Given the uncertainty of this charcoal’s context of origin, its inconsistency with the archaeological material discovered and the dates obtained elsewhere, this date has not been retained. The second charcoal, however, provided a date very close to those from the fireplace, at 9397−9028 calBP (ETH-113981). It was collected in square E1, thus near the fireplace, but in the upper part of the layer (z = −175 cm). Together, these four dates chronologically place layer 2 and its associated archaeological material in the Early Holocene, at the end of the 10^th^ millennium calBP.

#### Layer 1.

Layer 1, finally, is indurated and extremely compact. Grain size sample #3, collected from this layer, also shows this predominance of coarse silt (approximately 25%) and fine sand (approximately 26%) observed for layer 2, characteristic of regional wind-blown sediments (Table 4). The 16% of fine gravel distinguishing this sample from those of layer 2 correspond to centimeter-sized red pisolites and pisolites fragments. Some millimeter-sized black ferro-manganic nodules are also present in the layer. The association of these different elements indicates that layer 1 consists of a wind-blown formation that underwent colluvial evolution on a glacis and then pedogenesis. Thus, layer 1 can be interpreted as a pedo-sediment that served as a base for the development of the human activities recorded in layer 2.

This layer is archaeologically sterile, except for some rare small quartz flakes. Although it has not been dated, its position relative to layer 2 dated to the beginning of the Holocene suggests it is probably Pleistocene.

### Phytolith analysis

**Fig 5:**
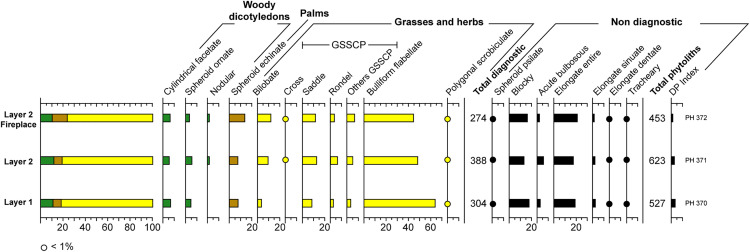
Summary of phytoliths recovered from the three RBX-1 samples. Sample PH 370 comes from layer 1, in the knapping workshop area (grain size sample #3). Sample PH 371 comes from the knapping workshop area of layer 2 (squares F1/G1, grain size sample #2), while sample PH 372 comes from the fireplace in layer 2 (square E1, grain size sample #1).

The three samples tested for RBX-1 (Fig 5) present enough diagnostic morphotypes (between 274 and 388). The results for the three samples indicate relatively open vegetation landscapes, as phytoliths produced by herbs and Poaceae largely dominate, reaching between 76% and 81% of diagnostic phytoliths. The bulliform flabellate morphotype produced in Poaceae and herb leaves is well-represented in all three samples (44–64%), suggesting a well-developed grass cover. These leaves notably contribute significant organic matter to the soils. Phytoliths produced by woody dicotyledons represent between 10% and 12%, while morphotypes representative of palms (Arecaceae) reach between 7% and 13%.

However, more marked similarities can be identified between the two samples from layer 2 (PH 371 and 372), particularly with the presence of nodular morphotypes produced by certain woody trees [74], the gsscp bilobate morphotypes, mainly produced by the Panicoideae subfamily and indicating mesophilic vegetation, and the gsscp cross morphotypes, produced by Poaceae and adapted to mesophilic to aquatic environments [36].

In the layer 1 sample (PH 370), the gsscp produced by Poaceae are dominated by the saddle morphotype, corresponding to the Chloridoideae subfamily and thus to vegetation more adapted to dry, xerophilic conditions [36].

The composition of these three samples reflects a vegetation type of open savanna with scattered trees, shrubs, and palms, with a dense grass cover. The morphotypes from layer 2 testify to more humid conditions, coinciding with the onset of the African Humid Period at the beginning of the Holocene. The relatively dense vegetation cover during this period may have favored pedogenesis and helped protect the sediment from erosion, thus contributing to the good preservation of the archaeological occupation of layer 2. Layer 1, in contrast, presents morphotypes characteristic of dry conditions, confirming its belonging to a more arid Pleistocene phase, likely characterized by a subsequent fersialitic pedogenesis favorable to the development of ferro-manganese concretions, as observed for this period on other sites in the Falémé valley [21].

Lastly, microcharcoals are logically much more numerous in the fireplace sample from layer 2 (PH 372), with nearly 300 counted, compared to 0 and 7 in the two other samples.

### Anthracological analysis of fireplace charcoals

**Fig 6:**
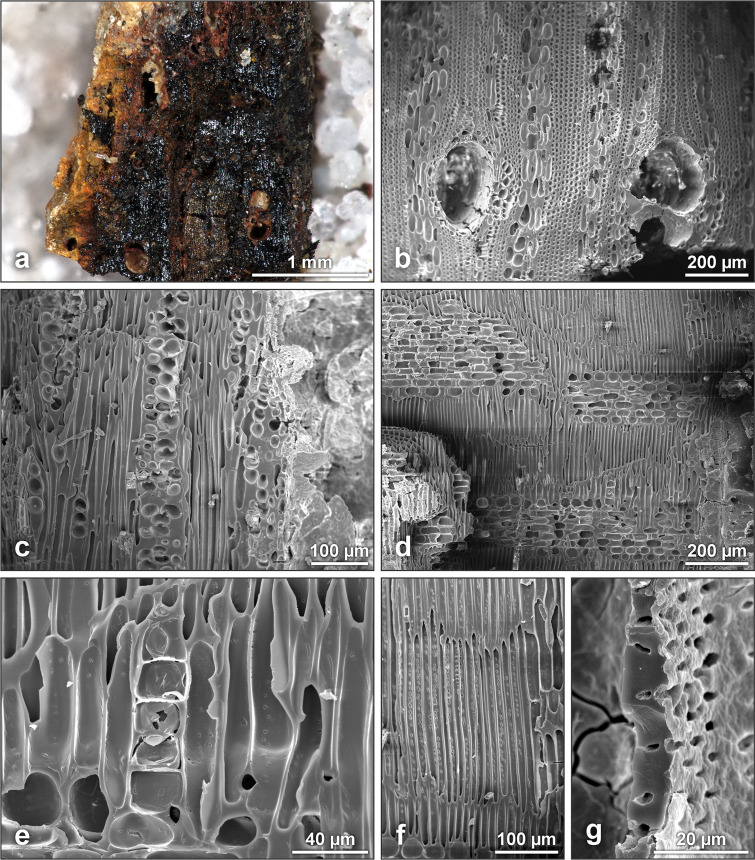
detarium spp. (a) Charcoal fragment with iron (hydro-)oxide concretions (from sample #A). (b-g) SEM images of a charcoal fragment (from sample #C). (b) Transverse section, short aliform parenchyma, fibers in a distinct pattern due to regular intrusive growth. (c) Tangential section, 3-seriate rays. (d) Radial section, homocellular rays of short procumbent cells. (e) Chambered axial parenchyma, formerly with prismatic crystals. (f) Distinct pattern of fibers in radial section. (g) Small vestures in intervessel pits.

In total, 64 fragments were analyzable: 30 from sample #A (inside of the fireplace), 25 from sample #B (top of the fireplace) and 9 from sample #C (outside of the fireplace) (Table 1). Among them, four fragments from the fireplace (samples #A and #B) were unidentifiable. The remaining 60 fragments all belong to the same charcoal type that was identified as detarium spp. Despite the iron (hydro-)oxide concretions hampering the anthracological identification, the following features were visible in all fragments: vessels solitary and in groups of regularly 2–3 vessels, vessel diameters often larger than 150 µm, regular intrusive growth of the fibers and relatively short procumbent ray cells. Further features, though not visible in all fragments, were the presence of axial canals in the transverse section and commonly 3- to 5-seriate rays. Common features of Fabaceae, such as prismatic crystals in chambered axial parenchyma, were only evidenced by means of Scanning Electron Microscopy (SEM), while vestures in inter-vessel pits were still not unambiguously discernible (Fig 6).

This identification supports the existence of a more humid savanna type as indicated by the phytolith analysis. Both savanna species of the genus detarium, *Detarium microcarpum* and *Detarium senegalense*, are present in Sudano-Guinean vegetation types. Ecologically, the dry savanna woodland species, *Detarium microcarpum*, is the more probable match; this shrub or small tree grows irregularly but is locally common in Guinean and Sudanian savannas, dry forests and fallows, and is common on ironstone soils; its wood is suitable as firewood [75,76].

The uniformity of the charcoal assemblage does not speak for a repeated or long-term use of the fireplace, unless it had been cleaned thoroughly after each fire event – which seems unlikely given the fact that the fireplace is very sparsely arranged and therefore does not seem to have been the object of recurrent and specific maintenance – or wood of the same species was consistently selected. As for the few fragments of detarium spp. from sample #C, they were probably translocated outside of the fireplace, imaginably by means such as wind or trampling.

### 1 Lithic material from layer 2

#### 2 General typo-technological overview.

**Fig 7:**
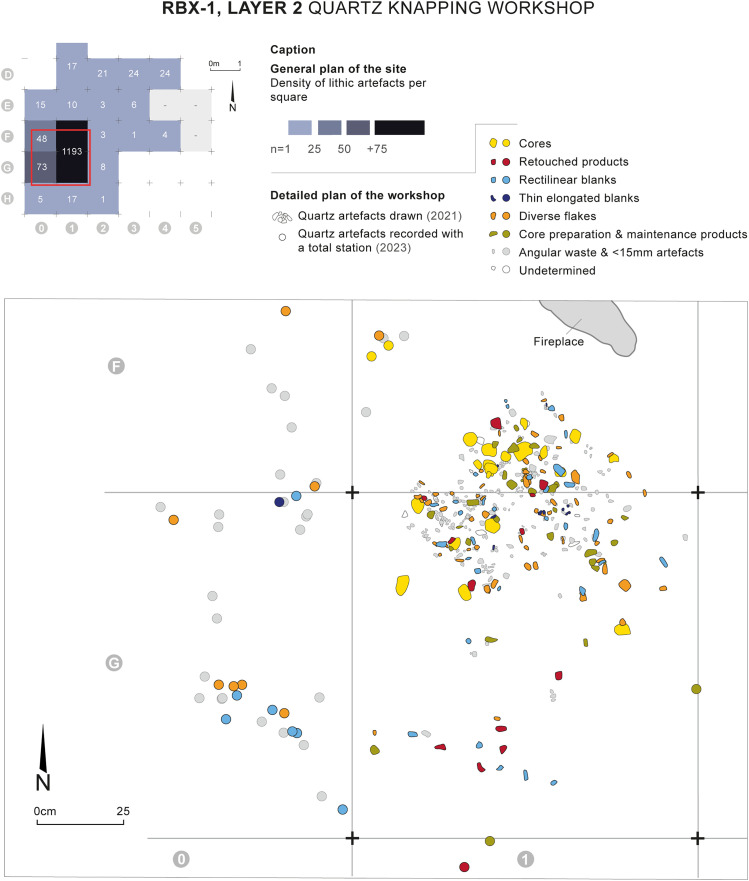
Spatial distribution of lithic material found in layer 2 of RBX-1 and typo-technological categories of lithic material collected in the quartz knapping workshop area. Drawing: C. Pruvost. CAD: E. Gutscher and C. Pruvost.

1,473 lithic pieces were discovered during the excavation of RBX-1’s layer 2, with 1,193 found within the knapping workshop (Fig 7 and Table 5). Among these 1,473 pieces, 636 were coordinated in situ using a total station, while the remaining 837 were collected through sieving. Objects whose technical and morphological characteristics did not allow repositioning into determined reduction sequences were excluded from the detailed typo-technological study. These were primarily angular waste and small flakes or flake fragments, representing nearly 80% of the assemblage (n = 1,184). Such a proportion is not surprising in a knapping workshop context.

**Table 5:** Typo-technological categorization of lithic material from layer 2 of RBX-1.

TYPO-TECHNOLOGICAL CATEGORIES			WORKSHOP		ELSEWHERE IN LAYER 2					TOTAL
			Quartz	Chert	Quartz	Alluvial quartz	Chert	Greywacke	Sandstone	
**STUDY CORPUS**	Cores		19	–	5	–	–	–	–	**24**
	Unretouched blanks	Flakes	113	–	38	1	1	–	1	**154**
		Elongated flakes	33	–	7	–	–	–	–	**40**
		Blades	9	–	8	–	–	–	–	**17**
		Bladelets	7	–	2	–	–	–	–	**9**
	Retouched products		16	–	4	2	–	–	–	**22**
	Pebble fragment with traces of bipolar percussion on anvil		–	–	–	1	–	–	–	**1**
**Subtotal of the study corpus**			**197**	**0**	**64**	**4**	**1**	**0**	**1**	**267**
**MATERIAL EXCLUDED FROM THE STUDY**	Angular waste		608	1	86	3	–	1	–	**699**
	Flakes	< 5 mm	–	–	2	–	–	–	–	**2**
		< 10 mm	17	–	9	1	–	–	–	**27**
		< 15 mm	20	–	5	2	–	–	–	**27**
	Flake fragments	< 5 mm	7	–	12	–	–	–	–	**19**
		< 10 mm	148	–	32	–	1	–	–	**181**
		< 15 mm	184	–	40	2	–	–	–	**226**
	Natural pieces		–	–	–	1	1	–	1	**3**
**Subtotal of the material excluded from the study**			**984**	**1**	**186**	**9**	**2**	**1**	**1**	**1184**
Undetermined			11	–	6	2	1	2	–	**22**
**TOTAL**			**1192**	**1**	**256**	**15**	**4**	**3**	**2**	**1473**
			**1193**		**280**					

The corpus selected for detailed study (n = 267) consists essentially of blanks (flakes, elongated flakes, blades, and bladelets; n = 242 in total), of which 22 show certain or probable traces of retouch. 24 cores are also present, with 19 found within the knapping workshop (three of these are core fragments).

**Fig 8:**
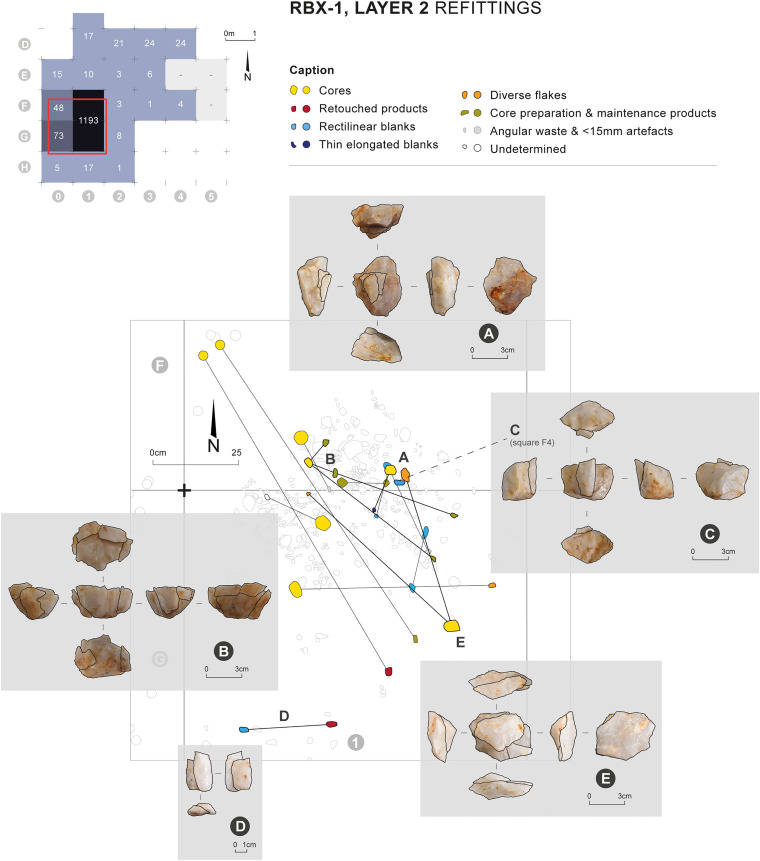
Refittings identified during the study of the material found in layer 2 of RBX-1. Drawing and photos: C. Pruvost. CAD: E. Gutscher and C. Pruvost.

The majority of the refittings made during the study came from the knapping workshop area. A total of 13 refitting sets were identified, involving 32 pieces in all, and nine of these sets are associated with cores (Fig 8). These nine sets are mostly composed of preparation or maintenance items that refit on their cores (cortical pieces, debordant flakes, etc.). A few refittings involving sought or even retouched products have also been identified. One refitting outside the knapping workshop is worthy of note: blade no. 19, found in the workshop, refits with core no. 543, found in square F4, about three meters to the east of the workshop (Fig 8, refitting C).

#### Raw materials.

The primarily exploited material at RBX-1 is quartz, representing over 99% of the material discovered in layer 2 and nearly 98% of the total mass of the assemblage (Table 6). Chert, sandstone, and greywacke are also present in negligible quantities, representing together less than 1% of the assemblage and approximately 2% of the total mass.

**Table 6:** Distribution of RBX-1 layer 2 raw materials.

Raw materials	n	% n	Mass (g)	% Mass
**Quartz (all combined)**	**1463**	**99.4%**	**1461.2**	**97.6%**
Quartz, type Q1	31	2.1%	15.7	1.0%
Quartz, type Q1-2	151	10.3%	299	20.0%
Quartz, type Q2	750	50.9%	1009.4	67.4%
Quartz, type Q3	29	2.0%	77.2	5.2%
Quartz, type Q4	2	0.1%	13.8	0.9%
Quartz, type Q5	1	<0.1%	8.7	0.6%
Quartz, undifferentiated	499	33.9%	37.4	2.5%
**Other materials**	**10**	**0.6%**	**35.7**	**2.4%**
Chert	5	0.3%	18.8	1.3%
Greywacke	3	0.2%	11.1	0.7%
Sandstone	2	0.1%	5.8	0.4%
**Total (n)**	**1473**	**100%**	**1496.9**	**100%**

Except for a chert angular waste discovered in square G1, the entirety of the artifacts constituting the workshop were knapped from a relatively homogeneous quartz. Its microcrystalline structure, slightly irregular/almost smooth fracture surfaces, and normalization of numerous products suggest good knapping aptitude [55]. Some variations were observed, particularly in the opacity and nature of the crystals. Consequently, three types of quartz were distinguished: Q1, Q2, and Q3 (Tables 6 and 7). These types are not always easy to distinguish, especially for small or thin pieces, which always appear more translucent due to their reduced thickness. Such pieces were grouped in the “undifferentiated quartz” category. Some pieces are situated at the interface of these types, with some being completely translucent at one end and opaque at the other (the “Q1-2” type). A continuity exists between these different quartz types, which seem to reflect intra-deposit and intra-block variability. We could therefore consider these different categories (Q1, Q2, Q3 and undifferentiated quartz) to be arbitrary, rather than representative of truly distinct quartz varieties from different deposits. The difference in proportion between the number of artifacts belonging to category Q2 (67.4%) and to categories Q1 and Q3 for example (respectively 1% and 5.2%) could be explained by a preferential selection of Q2 raw material blocks, to the detriment of the others. However, the artifacts produced on these different categories are identical overall, and there is no real evidence to support a desire for differential selection. It seems more likely that the difference in proportions is of a geological nature, reflecting the difference in crystal transparency within the vein. In any case, the continuity between these different categories attests to the high degree of homogeneity of the quartz selected for knapping at the site.

**Table 7:** The different types of quartz identified at the RBX-1 site.

Type	Structure	Crystals (color)	Crystals (transparency)	Break surface	Brilliance	Transparency
Q1	Microcrystalline	–	**Transparent**	Slightly irregular	Shiny	**Translucent**
Q1-2	Microcrystalline	White	Opaque and transparent	Slightly irregular	Shiny	**Opaque AND translucent**
Q2	Microcrystalline	White	Opaque and transparent	Slightly irregular	Shiny	**Semi-translucent**
Q3	Microcrystalline	White	**Opaque**	Slightly irregular	Shiny	**Opaque**
Q4	**Macrocrystalline**	White	Opaque and transparent	**Irregular**	Shiny	**Opaque to semi-translucent**
Q5	**Macrocrystalline**	–	Transparent	**Irregular**	Shiny	**Translucent**

In **bold**, the criteria that distinguish them from one another.

Two additional quartz types (Q4 and Q5), very different from the ones mentioned before, and characterized by a macrocrystalline structure and irregular fracture surface, were identified outside the knapping workshop, although their presence on the site is very anecdotal (n = 3).

The great majority of quartz exploited on the site appears to have been collected in the form of angular blocks resulting from the dismantling of veins. The natural surfaces observed on this material consist of a slightly pronounced and developing neo-cortex, creating irregular surfaces with a slightly smoothed appearance suggesting exposure to runoff. These surfaces are scattered with brown-orange inclusions, likely related to the surrounding lateritic sediments.

These altered natural surfaces suggest raw material collection either in primary position (directly from the quartz vein, with the neo-cortex representing the exposed and altered vein surface) or in sub-primary position (blocks detached from the vein but with limited transport and minimal fluvial alteration). Quartz veins are numerous in the Falémé valley, particularly on the east bank, and are still abundantly exploited for gold mining [77]. While the precise source of the blocks knapped in the RBX-1 workshop cannot be determined, these various indicators suggest a local supply, with materials transported to the site in the form of pluricentimetric blocks: the largest core in the assemblage (no. 202) measures approximately 57 mm in its greatest length.

Pieces presenting these natural surfaces are relatively few in the assemblage (n = 87; 5.9%), and among these are numerous angular waste and pieces less than 15 mm in size (n = 34). This low proportion of “cortical” elements can be explained by several possible scenarios:

• If collected in primary position, the selected angular blocks might have already presented few altered natural surfaces (vein outcrop surfaces), with part of the block’s surface remaining “fresh” and non-altered. In this case, debitage could have begun on these fresh fracture surfaces, producing few cortical elements.
• If collected in sub-primary position, the selected angular blocks might have been entirely or almost entirely neo-cortical. Nearly half the cores in the assemblage present areas of natural surfaces (n = 10); these were not entirely decorticated before exploitation, limiting the number of cortical elements present on the site.
• It must also be considered that some blocks might have been roughly shaped during collection, or at least before being knapped on the site.

More anecdotally, quartz pebble fragments are found outside the knapping workshop, with perfectly rolled neo-cortical surfaces testifying to their alluvial nature (n = 15, Table 5). The three pieces of types Q4 and Q5 display this pronounced alluvial neo-cortex. Quartz pebbles are abundant in the riverbed and on its banks, and are frequently exploited by Paleolithic knappers in the valley [22,28,29,78]. Chert, greywacke, and sandstone are also raw materials that locally outcrop, meaning no material present on the site has an exogenous origin [29,79].

#### Debitage methods and productions.

Quartz blocks in layer 2 were exploited exclusively through debitage. They were knapped by direct freehand percussion, presumably with a hard mineral hammer, as quartz is itself a particularly hard rock that is difficult to knap with a softer hammer. Nevertheless, archaeological evidence exists of using soft hammers for quartz, primarily for retouching or shaping activities, but not exclusively. Unfortunately, these two types of hammer are difficult to distinguish in terms of markers on quartz artifacts [53,54,80], and no hammer was found in layer 2. Only one piece with bipolar percussion on anvil traces was identified within the assemblage. This piece consists of a pebble fragment with four adjacent bidirectional negatives, associated on at least one of the “poles” of the pebble with traces of percussion/crushing.

Two primary types of sought products were identified on the site: blanks with straight, parallel edges, relatively wide and often thick, and elongated, narrow, and thinner blanks (Tables 8 and 9). These two types of blanks appear to have been obtained from two distinct reduction sequences, identified through the recurrence of these blank types in the assemblage and their scars on certain cores. The study of these different indices (products and cores), combined with refitting, revealed knapping modalities and a general organization of the cores that vary between productions, leading us to consider them as two distinct debitage schemes. However, certain pieces from these two reduction sequences occasionally share common characteristics, and some cores display scars belonging to both sequences (Table 10). This overlap of intentions necessarily impacts the precise assignment of products and by-products to each sequence. Furthermore, the limited number of whole products for each blank category (19 rectilinear blanks, 3 thin and elongated blanks, 66 diverse flakes, Table 8), although perhaps reflecting the selection and collection of sought blanks by the knappers after production, may induce biases concerning the representativeness of the assemblage. The hypotheses for reconstituting the reduction sequences discussed below, although based on multifactorial observations, must therefore be considered accordingly. Additionally, a wide variety of flakes is present in the assemblage, likely issued from diversified reduction sequences, with some probably being by-products of the main productions (Tables 8 and 9).

**Table 8:** Morpho-dimensional characteristics of the main productions identified within layer 2 of RBX-1.

Characteristics		Rectilinear blanks	Thin, elongated blanks	Diverse flakes
n		55 (of which 19 whole)	11 (of which 3 whole)	130 (of which 66 whole)
Length (mm)	Min.	18.37	17.40	9.57
	Max.	40.96	20.57	39.26
	Mean	27.05	20.56	21.84
Width (mm)	Min.	8.93	4.55	6.44
	Max.	28.33	12.10	37.86
	Mean	14.81	8.15	17.21
Thickness (mm)	Min.	1.79	1.55	1.6
	Max.	12.03	5.21	11.06
	Mean	4.46	2.75	4.83

We have only considered blanks that clearly belonged to one of the two productions. Length was only considered for whole pieces.

**Table 9:** Platforms of RBX-1 layer 2 cores and blanks, when preserved.

Sequence	Rectilinear		Thin, elongated		Flakes		Cores with diverse blanks	Other cores	Total
Platform type	Blanks	Cores	Blanks	Cores	Blanks	Cores			
Smooth/ Plain	12	4	3	1	34	–	2	5	**61**
Dihedral	5	4	–	–	16	–	1	–	**26**
Faceted/ Prepared	13	1	–	3	13	4	2	2	**38**
Linear	1	–	–	–	2	–	–	–	**3**
Punctiform	1	–	–	–	2	–	–	–	**3**
Crushed	–	–	–	–	2	–	–	–	**2**
Natural	2	1	–	–	8	–	1	–	**12**
Undetermined	2	–	–	–	8	–	–	–	**10**
**Total preserved platforms**	**36**	**10**	**3**	**4**	**85**	**4**	**6**	**7**	**155**
**Total artifacts by category**	**55**	**8**	**11**	**3**	**130**	**1**	**4**	**5**	**217**
	**63**		**14**		**131**				

Considering that a core can have several striking platforms, a total of 31 platforms were counted for the 21 whole cores discovered in layer 2. Three core fragments have not been included in this table.

**Table 10:** Morpho-dimensional characteristics of RBX-1 layer 2 cores.

Core characteristics		With rectilinear blanks	With thin, elongated blanks	With flakes	With diverse blanks	Other cores
n		8	3	1	4	5
Height of the debitage surface (mm)	Min.	29.22	37.50	39.83	21.75	12.61
	Max.	42.03	58.11	–	48.19	44.81
	Mean	36.55	45.38	–	37.59	27.56
Width of the debitage surface (mm)	Min.	35.76	44.18	53.40	41.65	18.83
	Max.	55.04	45.79	–	48.46	55.92
	Mean	45.09	45.04	–	45.26	39.89
Thickness of the core (mm)	Min.	17.36	12.07	11.83	19.63	5.55
	Max.	25.27	19.25	–	23.43	33.75
	Mean	20.37	15.66	–	21.94	15.82

Three core fragments have not been included in this table.

The preparatory phases of the different identified reduction sequences were difficult to document. Indeed, as mentioned in the “Raw materials” sub-section, only a limited number of pieces with natural surfaces are available to reliably inform on these preparatory phases (n = 87, 34 of which are angular debris or flake fragments smaller than 15 mm). The cores themselves were mostly abandoned at advanced stages of exploitation and therefore exhibit few, if any, flake scars from these early phases. When such scars are present, they have often been heavily reworked by subsequent removals, making them difficult to describe and interpret. Nevertheless, the standardization of the sought products suggests that particular attention must have been given to these preparatory stages, even though only limited evidence of them remains today. It should also be considered that by-products resulting from preparatory phases (but not necessarily cortical ones) are most likely present within the workshop, although they may not have been identified as such during the analysis. The wide variety of flakes discovered at the site certainly includes elements from the preparatory phases.

##### Production of rectilinear blanks

**Fig 9:**
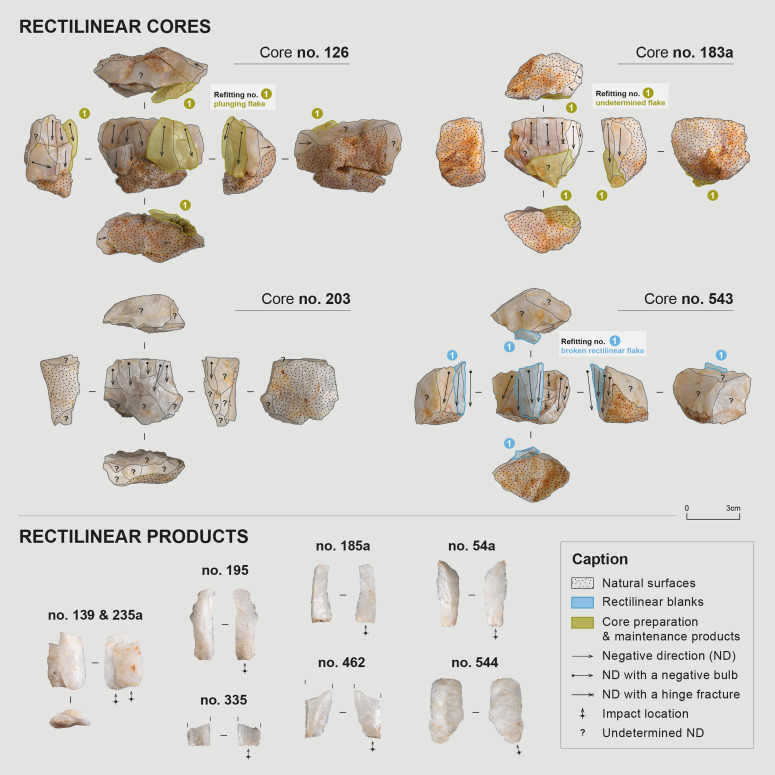
Material associated with the production of rectilinear blanks. Photos and CAD: C. Pruvost and E. Gutscher.

Elongated or non-elongated blanks with straight edges parallel to the debitage direction, and often with similarly oriented ridges, are designated here as “rectilinear blanks”. 55 of them were identified within the assemblage, although this count may not be exhaustive given the high degree of fragmentation at the site and the occasional difficulty in distinguishing blanks originating from the two main reduction sequences. Scars of such blanks were identified on 12 cores – among which 8 cores were used to produce exclusively this type of blanks (Tables 8 and 10). Seven refitting sets related to this reduction sequence were identified (Fig 9). From a typo-dimensional perspective, identified rectilinear blanks consist of flakes (n = 31), elongated flakes (n = 11), and blades (n = 13). It should be remembered, as mentioned earlier, that quartz knapping tends to produce numerous distal snap terminations [53–55,80], so much so that it can be difficult to determine whether this termination is related to a hinge accident, a post-knapping break or even whether the piece is whole and its abrupt termination is related to the morphology of its core. Nevertheless, scars of these different product types sometimes coexist on the same core and were likely extracted during the same sequence and with the same modalities. It would therefore seem that the primary characteristic sought in this reduction sequence scheme was the straightness and parallelism of the edges rather than the length of the blanks. The same applies to thickness: a portion of rectilinear products displays a marked thickness, reaching 12 mm, which is more than the thickest flakes in the assemblage (max. 11.06 mm) and significantly more than products from the second reduction sequence of elongated and thin blanks, the thickest of which measures only 5.21 mm (Table 8). However, some rectilinear products are quite thin (min. 1.79 mm). The gradual exhaustion of the lateral convexities at the end of the core’s exploitation, or even between two maintenance phases, may lead to variations in product thickness and explain this situation.

Cores displaying rectilinear product scars were mainly exploited using volumetric modalities to prepare and maintain convexities. They present a semi-rotating exploitation surface, most often unique and exploited unidirectionally. The striking platform is sometimes left natural or more often prepared thanks to one or a few large removals, resulting in essentially smooth platforms, occasionally dihedral. However, examination of the butt of rectilinear products suggests that the striking platforms were frequently, though not systematically, prepared by faceting the platform surface and by marked abrasion of the overhang (Table 9). Given that the cores were not prepared prior to their abandonment, the observed difference between the platforms on the cores and the butt types on the products is not inconsistent. Surfaces opposite the striking plane and the debitage surface are generally left natural or partially affected by removal during initial block preparation.

Two primary morphologies of cores with rectilinear products can be distinguished: “pyramidal” cores (n = 2) and “parallelepiped” cores (n = 5). Pyramidal cores present an exploitation surface higher than wide, with longitudinal convexities tapered in the distal part, giving them a “pointed” morphology (Fig 9, no. 183a). Some products from these cores consequently have a curved profile. Parallelepiped cores present an exploitation surface wider than high, with a rectangular morphology (Fig 9, no. 126, 203 and 543). Their longitudinal convexities are barely pronounced, so that the distal intersection of the exploitation surface presents a sharp angle. Products from these cores have a straighter profile than those from pyramidal cores and sometimes retain a portion of natural surface at their distal end.

On several cores, the negative bulbs of rectilinear products are missing, suggesting platform rejuvenation at some point during debitage. However, no rejuvenation tablets were identified in the assemblage. Such an action would shorten the core’s exploitation surface, making it possible that some parallelepiped cores originally had a more pyramidal morphology. Some cores indeed have a morphology intermediate between these two types (n = 3). Thus, rectilinear blades might have been obtained on pyramidal cores/ during early exploitation, while elongated flakes and then flakes were extracted on parallelepiped cores/ later in debitage. The cores were therefore not necessarily abandoned when block dimensions no longer allowed obtaining sufficiently elongated blades or flakes.

A continuity between these two core types is thus conceivable. Their maintenance methods are similar: their lateral convexities are maintained throughout the sequence by extracting debordant products (i.e., a product extracted at the edge of the core to enhance the lateral convexities), whose scars are visible on several cores and whose refittings confirm this interpretation (Fig 9). These debordant elements are produced in continuity and alternation with rectilinear products: they are themselves straight, although their triangular section, or the fact that the “debordant” surface is sometimes entirely neo-cortical, clearly differentiates them from sought rectilinear products. Nevertheless, if the straightness of products was the primary characteristic sought during knapping, it cannot be excluded that certain debordant products might have been selected for further use, following objectives like those of the rectilinear products. Some cores also bear the scars of debordant products that were not found in the assemblage; these elements may have been part of the products selected by the knappers after their extraction.

Several cores (n = 6) present one or multiple hinge fractures on their exploitation surface (Fig 9, no. 126, 183a and 543). It is interesting to note that certain flakes found in the assemblage seem to testify to a desire to clean these accidental surfaces to pursue debitage. These flakes are thick, plunging, and bear on their dorsal face the scars of one or multiple hinge fractures, suggesting that the percussion to extract them was delivered further back, inside the striking platform, to eliminate the accidental surface.

The exhaustion of the lateral convexities and hinge fractures appear to be the primary reasons for abandoning these cores with rectilinear products. In both cases, the reduced dimensions of cores at the end of exploitation (max. height 42.03 mm, max. width 55.04 mm, max. thickness 25.27 mm; Table 10) might no longer have allowed implementing the previously adopted maintenance solutions, such as removing new debordant products or cleaning accidental surfaces.

Some cores nonetheless present “serial” hinge fractures – an inevitable phenomenon when a hinge fracture already exists on the exploitation surface and the blow is not struck sufficiently far back to clean the accident. The fact that this situation was observed on several cores raises questions about intentionality, particularly when the straightness of edges rather than blank length is prioritized. Indeed, for blocks with a reduced raw material reserve that could not be exploited for much longer, continuing to extract blanks despite knapping accidents would allow maximizing the core’s potential.

##### Production of elongated, narrow, and thin blanks

Thinner products than the previously described rectilinear products also seem to have been sought. These are generally elongated and narrow, consisting essentially of bladelets or elongated flakes. These products are relatively standardized, and while a certain regularity can be observed, the delineation of their edges and ridges is less straight and parallel than those described as “rectilinear”.

**Fig 10:**
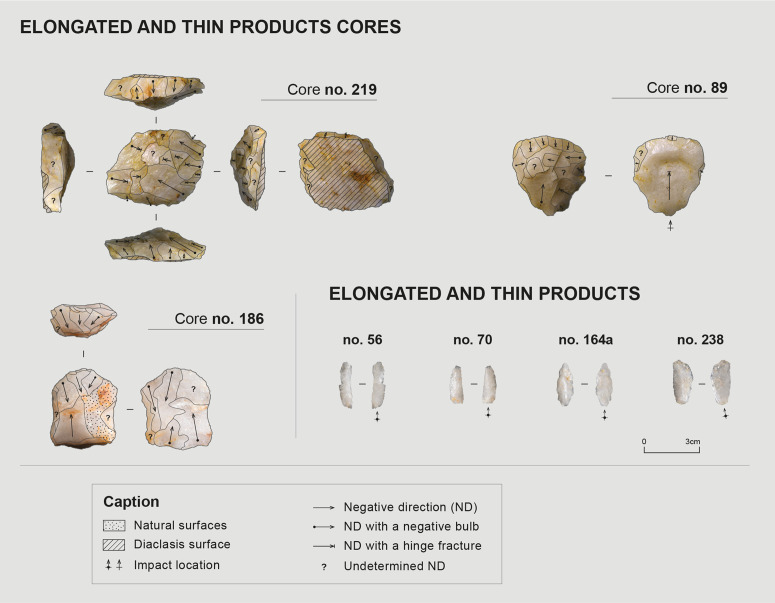
Material associated with the production of elongated, narrow, and thin blanks. Photos and CAD: C. Pruvost and E. Gutscher.

When examining the scars of these products on cores and certain blanks in the assemblage (Fig 10), one observes that they frequently present an oblique distal termination, generally lateralized to the left. These blanks appear to have been systematically detached from equally oblique striking platforms, resulting in oblique butts. By striking slightly offset from a guiding ridge, the shock wave’s propagation from an oblique striking platform can create an asymmetry during blank detachment. The recurrence of these characteristics specifically for these products (the thick rectilinear products typically presenting a straight, or even snap, distal termination) suggests a deliberate intention to produce this specific morphology. Nevertheless, these blanks seem less standardized than the rectilinear products described earlier, making them more difficult to identify within the assemblage (Table 8, n = 11).

At least three cores present scars of such products (Fig 10). Their knapping modalities and general organization differ from cores with rectilinear products. These are hierarchical cores with two opposite surfaces, generally an exploitation surface and a striking platform or maintenance surface. For one of them, the striking platform surface was left partially natural (Fig 10, no. 186), while for another, it consists of a diaclasis surface (Fig 10, no. 219). Core no. 89 (Fig 10) is more difficult to interpret and is likely a flake core with a deep scar terminated in a hinge on its ventral face, which removed the flake’s platform. In all cases, these three cores show little evidence of platform preparation, except for a small and localized faceting. Among the blanks associated with this reduction sequence, only three have preserved butts, all of which are systematically smooth.

Two of the three cores were exploited bidirectionally to produce the elongated blanks, from two opposed striking platforms (Fig 10, no. 186 and 219). The third was also exploited from two striking platforms, this time orthogonal to each other (Fig 10, no. 89). Lateral convexities maintenance of the exploitation surface was achieved by detaching small, intersecting, and curved removals, perpendicular to the debitage direction.

The reasons potentially motivating the abandonment of these cores are multiple: two of them present “cascading” hinge fractures accumulated at the center of their exploitation surface, and several display internal fissures and a fragility of the raw material. A systematic exhaustion of convexities associated with reduced dimensions that make their maintenance difficult confirm that these cores were exploited until the possibilities of obtaining the sought blanks were exhausted.

##### Relationships between these reduction sequences

As previously mentioned, the two primary reduction sequences identified within this occupation are not always easy to distinguish from one another. While the sought blanks are substantially different, they nonetheless share a certain regularity and parallelism of edges, making the assignment of certain products to one sequence rather than another difficult. While most identified rectilinear blanks present a marked thickness, enabled by the volumetric production modalities of these blanks, this thickness directly depends on the state of lateral convexities of the core and the possibilities of their maintenance. Some thin blanks with rectilinear edges could therefore have been extracted during stages of debitage. The exhaustion of lateral convexities of a core can sometimes impact the straightness of product edges. It is thus not impossible that certain less “regular” products also originate from cores and sequences defined as “rectilinear”. This identification criterion is therefore not systematic, making it difficult to distinguish pieces with regular edges but low thickness.

**Fig 11:**
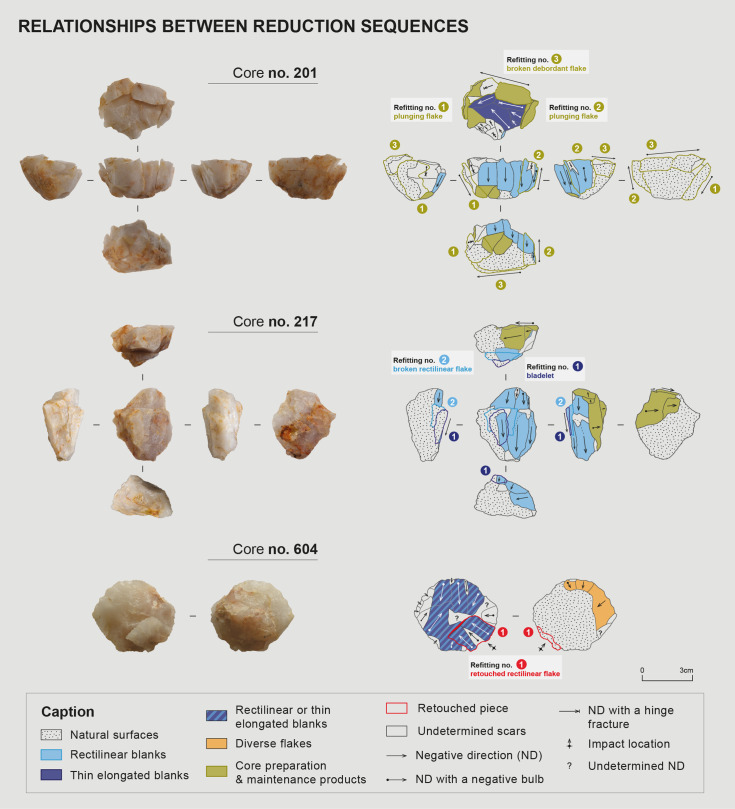
Cores used to produce different types of blanks. Photos and CAD: C. Pruvost and E. Gutscher.

Core no. 604 illustrates well the proximity between these two reduction sequences (Fig 11, no. 604). It is a hierarchical core with two opposite surfaces, an exploitation surface, and a striking platform surface left partially natural. It was exploited bidirectionally and underwent lateral convexity maintenance through small, intersecting, and curved removals. In this sense, it is extremely like the modalities described for the production process of elongated and thin blanks. However, a refitting was identified with a blank initially categorized as rectilinear: a relatively thick piece (5.39 mm) with straight, parallel edges and ridges. However, this piece presents an oblique distal termination – partially retouched – a characteristic primarily observed in the reduction sequence of elongated, thin blanks. The scars observed on the core also show mixed characteristics, with very regular, even rectilinear edges and oblique distal terminations.

This refitting, situated at the interface between the two proposed reduction sequences, could argue in favor of there being only one reduction sequence presenting a certain level of variability. The sought blanks would be more or less regular/ rectilinear, and the methods deployed to produce them would also be variable.

Similarly, refittings made on core no. 217 revealed that bladelets were extracted alongside the rectilinear blanks that constitute the main visible scars on the core (Fig 11, no. 217). This core corresponds exactly to the volumetric cores with pyramidal morphology described for the rectilinear product reduction sequence. Its lateral convexities are maintained by removing peripheral or even debordant products that enabled the extraction of thick rectilinear blanks. However, at least one thin bladelet was extracted within the same sequence as the other products. It is both posterior and anterior to the extraction of thick rectilinear blanks. Given that the thickness of rectilinear blanks varies according to lateral convexities exhaustion during knapping, such a situation is not so surprising.

Core no. 201 also presents scars associated with the two previously described blank types, but they are organized differently on the core (Fig 11, no. 201). Initially, this core was used to produce rectilinear blanks on a semi-rotating surface, maintained through the removal of debordant products. The surface that served as a striking platform for these blanks subsequently served, in a second phase, as an exploitation surface to extract elongated and thin blanks with oblique distal terminations. The initial exploitation surface for rectilinear products then served, in turn, as a striking platform. The refittings on this core confirm that the blanks produced during the two exploitation phases of the core are very different from one another: thick, rectilinear, and with straight distal terminations in one case; thin, elongated, less regular, and with oblique distal terminations in the other. Thus, the succession of these two exploitation modalities on two different surfaces, rather than their cohabitation within a single debitage sequence, tends to confirm the importance of distinguishing these two reduction sequences.

The products sought at RBX-1 seem to be situated on a continuum, with the two extremities represented by the two exploitation phases of core no. 201. The reasons for this variability can be multiple, ranging from the desire to produce distinct blank types for different functional objectives to a certain degree of adaptability to the raw material, to potential accidents and to opportunities arising during knapping. The same goes for the production methods of these blanks. Certain methods seem preferentially designed and particularly well-suited to the production of a specific type of blank. However, they can also be used to produce other types of blanks, within the same sequence or during successive knapping phases. In the latter case, as the various reduction sequences described do not require the entire block to be invested, they can be considered as additional structures that can be deployed on the same core when the latter no longer meets the criteria required for a specific production and/or meets the criteria required for another scheme [81].

##### Flake production

Flakes—apart from those considered among the rectilinear products—are numerous in the assemblage (n = 130, Table 8), and flake scars are visible on all cores, including those associated with the two reduction sequences described above.

These flakes are highly diverse, and a detailed study of them did not reveal any evidence of standardization. Overall, they exhibit greater dimensional variability than the products associated with the two main reduction sequences (min. length: 9.57 mm; max.: 39.26 mm; min. width: 6.44 mm; max.: 37.86 mm; Table 8). Their butt types, although predominantly smooth (n = 34), also include dihedral (n = 16), faceted (n = 13), linear (n = 2), punctiform (n = 2), crushed (n = 2), and natural (n = 8) types (Table 9). Regarding their distal terminations, when preserved, these are mostly feathered (n = 37), but a substantial number of flakes show snap fractures (n = 26), which are common on quartz artifacts (Table 11). In plan view, distal terminations are mostly straight (n = 32), though their overall morphology is highly variable.

**Table 11:** Termination types of RBX-1 layer 2 flakes.

Terminations (when preserved)		In cross-section				Total
		*Feathered*	*Step*	*Snap*	*Plunging*	
**In plan view**	*Straight*	8	4	16	4	**32**
	*Convex*	9	1	1	2	**13**
	*Pointed*	11	2	2	–	**15**
	*Left-oblique*	3	3	3	2	**11**
	*Right-oblique*	2	–	1	–	**3**
	*Complex*	4	1	3	1	**9**
**Total**		**37**	**11**	**26**	**9**	**83**

The distal termination of 47 out of the 130 flakes recovered from layer 2 was not preserved.

The scar patterns, for their part, provide useful information about the cores from which these flakes were detached (Table 12). Indeed, when determinable, the patterns were found to be mostly multidirectional (n = 44). Apart from the cores associated with the rectilinear reduction sequence—which are predominantly unidirectional (22 flakes are themselves unidirectional)—most of the other cores in the assemblage are either centripetal or multidirectional. We may therefore consider that most of the multidirectional flakes originate from various reduction sequences, excluding that of the rectilinear products. However, it cannot be ruled out that some of the multidirectional flakes derive from preparatory phases of different reduction sequences, including the one that ultimately produced rectilinear and unidirectional blanks. In any case, in both reduction sequences, flakes appear to be removed prior to the detachment of the sought blanks, whether rectilinear or elongated. Core preparation and maintenance would therefore be the only reason to extract flakes in these sequences.

**Table 12:** Dorsal scar patterns of RBX-1 layer 2 flakes.

Scar pattern	n	%
Unidirectional	22	16.9
Bidirectional (two opposite directions)	5	3.8
Perpendicular/ Orthogonal (two directions at a right angle)	11	8.5
Centripetal	1	0.8
Multidirectional	44	33.8
Undetermined	47	36.2
**Total**	**130**	**100**

**Fig 12:**
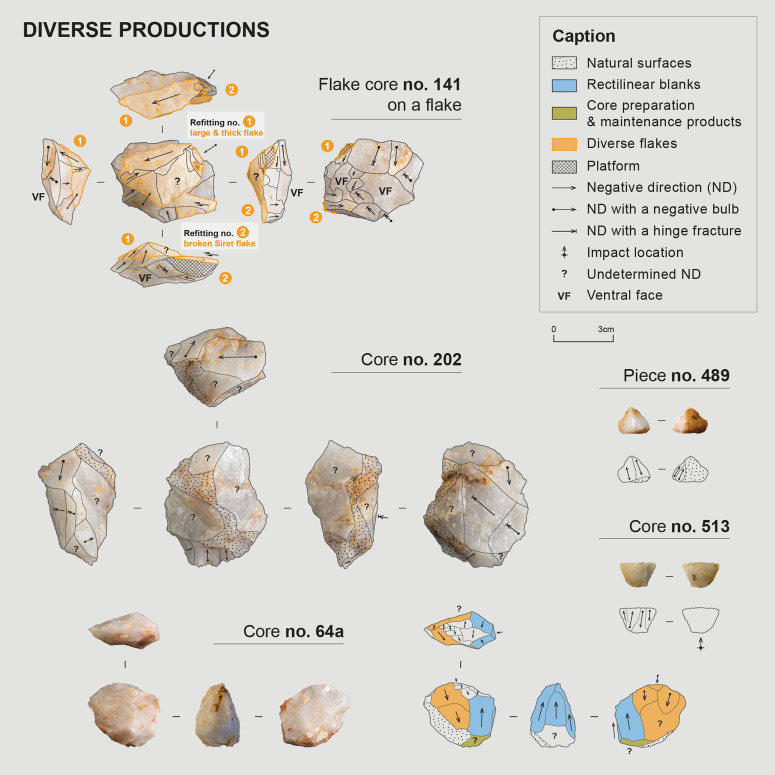
Flake cores, diverse cores, pebble fragment with bipolar percussion on anvil traces. Photos and CAD: C. Pruvost and E. Gutscher.

However, one core used exclusively to produce flakes has been identified in the assemblage (Fig 12, no. 141). Several large flakes were extracted from it, one of which, flake no. 66, was found within the workshop and refitted on the core (length: 39.26 mm; width: 29.16 mm; thickness: 10.72 mm). Originally quite thick, the convexities of this core appear to have been maintained by the removal of small intersecting flakes, in a manner like that described for cores with elongated, thin blanks. The detachment of flake no. 66 has exhausted the convexities of the block, most certainly leading to the abandonment of the nucleus, which is now only 11.83 mm thick and therefore difficult to exploit further.

In parallel, core no. 64a, initially used to extract a few rectilinear blanks, was later employed to produce several flakes (Fig 12, no. 64a). This core has two opposite main surfaces, either fully or partially prepared. One of its narrow faces, forming the intersection between the two main surfaces, was exploited to extract three rectilinear blanks from the same striking platform. The scars of the flakes removed in a second stage are all located along the same edge of the core, detached from both of its faces. Their dimensions are relatively small (max. length: 14.29 mm), raising questions about their function: were they scars of sought blanks, or were they detached to modify the core, specifically to create a convex sharp edge? It is difficult to decide between these two hypotheses, as the small size of the flake scars does not correspond to any phenomenon identified elsewhere in the assemblage, nor does the sharp edge’s typology, which has no equivalent among the retouched pieces. In the absence of a use-wear traces analysis, the interpretation of this core remains open.

The intention behind flake production at the site, in general, is difficult to grasp. Flake no. 66 and the other flake scars visible on the cores do not exhibit standardized morphology. Flake no. 66 was not retouched, and its presence within the knapping workshop suggests that it was probably not selected for later use. The great diversity of flakes found in the assemblage does not always allow for a clear distinction between those resulting from core preparation or maintenance and those that were sought blanks – two categories that are not necessarily mutually exclusive. However, if the need for flakes was only occasional and did not require them to be particularly standardized, this could explain the diversity of flakes and their scars in the assemblage, as well as the fact that only one of the 24 discovered cores was exclusively dedicated to flake production. According to this hypothesis, flakes extracted as part of the preparation or maintenance of rectilinear and/or elongated blank cores may also have been suitable for selection and use, thereby reducing the need for additional cores specifically dedicated to flake production.

##### Other productions

Several cores in the assemblage display a limited number of scars that do not follow a specific pattern, appearing therefore more “opportunistic” (n = 8, Table 10, and Fig 12). Six of them are very small and have highly irregular surfaces, making it difficult to interpret earlier stages of exploitation; three of these six are even core fragments.

The two remaining cores consist of: 1) A large block of fissured raw material of lower quality, on which a few negatives are visible but with few preserved negative bulbs (Fig 12, no. 202); and 2) A blank broken at the mesial part, whose snap fracture served as a striking platform for the extraction of two extremely short and thin bladelets (Fig 12, no. 513).

A fragment of a pebble found outside the workshop, in square D4, exhibits traces of bipolar percussion on anvil: multiple bidirectional negatives, a fracture initiated from two opposing points [80,82,83] (Fig 12, no. 489). This is the only evidence of the use of this technique within this occupation.

##### Retouched material

**Fig 13:**
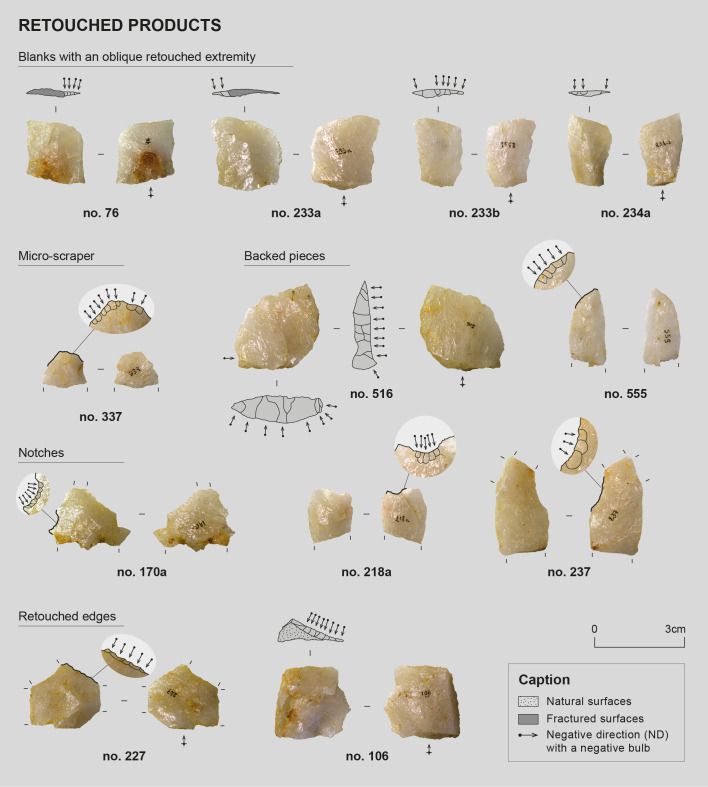
Retouched products. Photos and CAD: C. Pruvost and E. Gutscher.

Several retouched artifacts have been identified within layer 2 of RBX-1: 17 pieces with definite retouch and 5 with possible retouch. They mainly consist of retouched edges and notches; two backed pieces and a small scraper were also identified (Table 13 and Fig 13).

**Table 13:** Blanks selected to produce retouched elements.

Retouched products		Rectilinear blanks	Thin elongated blanks	Diverse flakes	Undetermined flake fragments	Total
Definite (n = 17)	Notch	1	–	–	4	**5**
	Retouched edge	5	–	3	1	**9**
	Backed piece	1	–	1	–	**2**
	Micro-scraper	–	–	–	1	**1**
Possible (n = 5)	« Clactonian » notch	–	–	1	–	**1**
	Multiple « clactonian » notches	1	–	–	–	**1**
	Retouched edge	1	1	1	–	**3**
**Total**		**9**	**1**	**6**	**6**	**22**

The notches (n = 5, Table 13 and Fig 13, no. 170a, 218a, and 237) all have a semi-abrupt angle, ranging between 60° and 68°. This retouch is most often inverse, meaning it was applied from the dorsal face of the blank and is visible on the ventral face (n = 4/5). Their extent is generally short (n = 4/5); the only piece with a long extent is also the one with the most acute angle (60°; Fig 13, no. 237). One notch was made on a rectilinear blank (Fig 13, no. 218a), while the other four were made on fragments of indeterminate flakes. Their location on the blanks varies: distal, proximal right, left edge. The same applies to the morphology and regularity of the retouch, which ranges from regular parallel and subparallel to irregular scalar retouch. This morphological diversity may be explained by the nature of the raw material, as quartz produces prismatic fracture surfaces that are more difficult to standardize. These are small notches, with an amplitude not exceeding 14 mm (min. 5 mm); however, the presence of multiple clearly identifiable retouch negatives leaves little doubt about their anthropogenic origin. Other notches are more ambiguous: these are the so-called “clactonian notches,” consisting of a single removal negative. Their dimensions are even smaller (approximately 2.5 mm in amplitude), and since they are rare (two pieces, one of which has three clactonian notches), it is difficult to determine their intentionality within this corpus.

Twelve pieces (including three uncertain cases) have a retouched edge, often only partially retouched (Table 13 and Fig 13). Among them, four rectilinear pieces exhibit a distal truncation, retouched to create an oblique termination, possibly to shape an asymmetrical point, systematically lateralized to the left (Fig 13, no. 76, 233a, 233b, and 234a). This configuration is reminiscent of the scars and unmodified products with oblique distal terminations described in the reduction sequence for elongated blanks, making it even more plausible that this characteristic was intentionally sought. This oblique retouch is always marginal, suggesting it was applied to a blank that was already more or less standardized and required only minimal modification. It is consistently abrupt (between 88° and 90°) and applied from the ventral face, making it direct retouch.

The other retouched edges display diverse characteristics and were likely not all used for the same function. The pieces with definite retouch can be described as follows:

• No. 106: a debordant flake with a short, direct, rectilinear, and abrupt (90°) retouch on its distal termination (Fig 13, no. 106);
• No. 227: a curved flake with a short, direct, oblique, and semi-abrupt (67°) retouch on the right side of its distal termination, while the left side is broken (Fig 13, no. 227);
• No. 457: a flake displaying a portion of neo-cortical alluvial surface and a Siret fracture, retouched obliquely on its distal termination. The retouch is short, direct, and abrupt (70°). This piece also shows a possible clactonian notch;
• No. 653: a mesio-distal fragment of a rectilinear elongated flake with a fine, short, direct, and semi-abrupt (68°) retouch on the right side of its distal termination, while the left side is broken.

A small scraper (with a front angle of 88°) was found within the workshop (Fig 13, no. 337). It was made on the distal termination of a blank broken in its mesial part, making it technically a flake. However, we cannot be certain that it was not originally an elongated flake or even a blade before breaking. Its current dimensions are 12.28 mm in length, 14.97 mm in width, and 4.42 mm in thickness.

Finally, the two backed pieces identified in the assemblage are very different from each other (Fig 13, no. 516 and 555). The first was made on a large flake (no. 516, length: 28.46 mm; width: 29.77 mm; thickness: 6.36 mm), while the second was made on a rectilinear blade, which was unfortunately broken in its mesial part during excavation, resulting in the loss of the proximal section (no. 555, width: 12.51 mm; thickness: 12.03 mm). The latter underwent only very localized and minimal modification: taking advantage of the already slightly convex delineation of the blade’s left edge, this convexity was accentuated on the distal termination to create a point (Fig 13, no. 555). The few applied retouch negatives are short, semi-abrupt (50°), and direct. As for piece no. 516, the selected flake has an enveloping butt on a semi-circular edge. This edge, running from the proximal to the distal part along the right edge, was likely used as a back, as suggested by the presence of multiple retouch negatives that helped refine its convexity (Fig 13, no. 516). These retouch scars are short, abrupt (90°), and direct. One of the extremities created by this back even appears to have been further modified by the addition of a small notch, giving it the morphology of a short point.

It is difficult to provide a clear interpretation of the intended purpose of knapping activities at this site based on the discovered tools. Retouched pieces are relatively scarce, and there are few recurring typological patterns among them. Their retouch is often short and marginal, and on quartz, its intentionality can sometimes be difficult to establish. Experiments were conducted following the discovery of quartz pieces with micro-notches at Sibudu Cave in South Africa [84]. These experiments demonstrated that such micro-notches—and even some edges that strongly resemble intentional retouch—can result from various factors, including technological accidents during knapping, taphonomic processes such as trampling, or functional wear from use as hafted armatures. The marginal nature of the retouch observed at RBX-1 should perhaps be considered in light of these findings.

Nevertheless, despite being fine, the retouch at RBX-1 is systematically deeper, more regular, and less superficial than the taphonomic chipping observed on some pieces in the assemblage (Fig 13, unretouched but chipped edges of piece no. 170a). Consequently, there is little risk of confusion regarding the intentional nature of the retouch classified as “definite”. The low number of retouched pieces may be related to the fact that this occupation represents a knapping workshop, as indicated by the abundance of manufacturing waste. Such a site function implies that blanks were knapped on-site but not necessarily used and discarded at the place of production.

Moreover, certain similarities, particularly in the morphology of some unmodified blanks and others with marginal retouch, suggest that significant attention was given to the preparation and maintenance of cores to produce blanks as standardized as possible. Another, non-contradictory, explanation could be that the blanks obtained through the reduction strategies employed by the knapper(s) at this site simply did not require extensive retouch. The presence of retouch that enhances features seemingly sought after from the initial blank extraction stage suggests an intentional modification. However, it remains difficult to be categorical when the retouch is so marginal, and the obtained products are typologically unstandardized. Use-wear trace analysis of the assemblage, further quartz knapping experiments, and comparisons with other quartz assemblages displaying similar characteristics are all necessary future avenues for a better understanding of this collection. In the absence of such studies, we refrain from drawing definitive conclusions and remain cautious in the hypotheses we formulate.

### Summary of lithic study results and interpretation of site function

The study of the lithic assemblage from layer 2 of RBX-1 confirms the initial hypothesis that we are dealing with a particularly well-preserved quartz knapping workshop. Indeed, the workshop is spatially very restricted and is essentially made up of knapping waste: cores, core maintenance products, angular waste, etc., whereas retouched products are relatively rare. The numerous refittings carried out throughout the study confirm our interpretation of the site’s function. The detailed typo-technological study of the material from the knapping workshop has highlighted raw material procurement strategies in a primary or sub-primary context, aimed at collecting high-quality, highly homogeneous quartz blocks. It appears that these blocks may have undergone significant investment in preparation and maintenance stages to produce two main types of standardized blanks: relatively wide and thick blanks with rectilinear and parallel edges, and elongated, narrow, and thin blanks with an oblique distal termination, angled to create a natural asymmetric point. Similarities exist between these two types of blanks, and occasional overlaps have been observed in some cores associated with these two reduction strategies, suggesting that the site’s production objectives lie along a continuum. Retouched products are scarce within the workshop. They mainly consist of notches and retouched edges, along with a micro-scraper and two backed pieces. Overall, these elements exhibit only marginal retouch, suggesting that the strong investment in blank standardization during the extraction stage reduced the need for subsequent modification.

The homogeneity of this assemblage and the exceptional consistency of the radiocarbon dates obtained from the fireplace suggest a single, brief occupation episode, a hypothesis further supported by the anthracological analyses. No discovered elements indicate a more permanent or recurrent occupation: 1) No postholes or stake holes were found, which would suggest a stable campsite; 2) No remains other than lithic artifacts and charcoal from the fireplace were recovered, which could have indicated diverse activities taking place at the site; 3) Only one knapping workshop was identified, and its highly homogeneous content does not suggest that it resulted from multiple knapping events occurring at different times in the same location. However, it is important to acknowledge that certain categories of remains are poorly preserved in West African sediments from ancient periods, potentially introducing a preservation bias. This is particularly true for bones; given that carbonization favors their preservation, we can assume that if the fireplace had been used for extensive cooking activities, faunal or carpological remains would likely have been found. Yet, in the absence of such remains, the occupation of layer 2 at RBX-1 appears to have been very brief and specialized in nature.

## Discussion

The interdisciplinary studies presented in this article establish the RBX-1 site as an important new reference point in the understanding of the West African Paleolithic. The following discussion aims to contextualize these results within a broader regional framework, first from a paleoclimatic and paleoenvironmental perspective and then from an archaeological one.

### Paleoclimatic and paleoenvironmental context at the Pleistocene-Holocene transition

The Early Holocene follows Marine Isotope Stage 2 (MIS 2; 29−14 ka), the final phase of the Pleistocene, characterized by highly unstable climatic conditions and marked by an arid optimum during the Last Glacial Maximum (LGM). This period, sometimes referred to in Africa as the “Big Dry” [85–87] or the “Ogolian” [88], is associated with occupation hiatuses in several regions of the continent, suggesting migratory processes that led human groups to abandon certain areas [86,87,89–92].

In West Africa, the long archaeological sequence from the Ounjougou complex (14.2°N), located along the Yamé river in Mali, is itself marked by a sedimentary and/or occupational hiatus during the LGM, following a period of extremely dense valley occupation during MIS 3 (57−29 ka) [8,9,93]. The lower Falémé valley in Senegal (13.5°N) offers a complementary sequence to this record. While MIS 3 sites are less numerous in this region, several sites indicate that the valley remained occupied during MIS 2 [10,94]. Geomorphological and geochronological studies conducted in the lower valley confirm that, far from drying up, the river remained active during this period, depositing nearly four meters of sediment [79]. Episodes of torrential flooding, characteristic of the climatic instability of the time, are even visible in natural profiles (e.g., C_SC_ layer at Toumboura) [79]. At a time when the southern margin of the Sahara desert potentially reached a latitude of 14°N [95], or even 13°N during Heinrich Stadials 1 (18.5–15 ka) and 2 (25.5–23.5 ka) [96], it is likely that the Falémé valley, with its active watercourse, provided favorable conditions and resources for human and animal habitation. The valley may thus have functioned as a refuge area, while other previously densely populated zones were eventually abandoned.

From 17 ka onward, geomorphological and paleoenvironmental studies in West Africa reveal alternating humid and arid phases, foreshadowing the progressive establishment of the more favorable climatic conditions characteristic of the African Humid Period (AHP) [93,97–100]. It is within this context that the first LSA industries of the Falémé valley were identified, dating to the very end of the Pleistocene. The oldest of these sites is Toumboura I (also referred to as Toumboura I-a and Toumboura I-2017), dated to 16 ± 1 ka [22,94,101].

Phytolith and anthracological analyses conducted at RBX-1 suggest arid, Pleistocene-like conditions during the formation of layer 1, whereas layer 2 reflects more humid conditions characteristic of the AHP, in agreement with the radiocarbon dates. The grain size analysis of layer 2 also supports the presence of sediments protected from runoff by a sparsely wooded but dense vegetative cover. These findings closely resemble those obtained from Fatandi V, another site in the Falémé valley roughly contemporary with RBX-1 [21]. The landscape in which the occupants of these two sites lived thus consisted of an open savanna dotted with trees, shrubs, and palms.

### Comparison with other LSA sites in West Africa

Despite a general trend toward microlithization of the toolkit, syntheses on the West African LSA generally agree that it is highly diverse, characterized by a mosaic of different technological behaviors [14,102,103]. The limited number of sites and their uneven spatial distribution make it difficult to establish a model explaining this diversity: Is it strictly chronological or geographical variability? Does it reflect differences in site function and habitat types? Or could it result from the coexistence of groups with distinct technical knowledge and traditions within the same territory? In this context, the assemblage from layer 2 at RBX-1 provides an opportunity to further explore these questions from a new perspective. How does this assemblage fit within the broader framework of the West African LSA? Are there significant similarities or differences in the technological behaviors adopted by the human groups who produced these assemblages? Do any regional and/or chronological trends emerge from these comparisons?

To address these questions, the discussion is organized into two parts: first, a comparison at the local scale, focusing on other LSA sites from the Falémé valley; and second, a broader comparison with sites from across West Africa (Table 14).

**Table 14:** Chronological summary of key characteristics of the main sites mentioned in the discussion.

Sites	Country, region	Dates		Paleoclimate and paleoenvironment	Raw materials		Typo-technological features				Other notable features	Reference(s)
		14C	OSL		Sources	Rocks	Tools	Knapping methods	Blanks	Technological features		
Njuinye	Cameroon	39,181–19,068 calBP	–	Guineo-Congolian forest	Local, secondary position	Lower phase: Quartz pebbles (exclusively)	Scrapers, drills, notches, geometric microliths	Freehand direct percussion	Flakes	Low standardization	Two phases, but technological similarity across phases; environmental and climatic stability	[4,5]
					Local	Upper phase: Quartz (dominant), but also obsidian, chalcedony, quartzite, etc.						
Shum Laka	Cameroon	38,406–6,081 calBP	–	Guineo-Congolian forest	Local	Quartz (dominant), basalt, tuff, obsidian	Segments, backed pieces, scrapers, borers	Freehand direct percussion; one core with traces of bipolar percussion on anvil	Flakes essentially, a few bladelets	A few discoidal cores	Technological continuity; environmental and climatic stability	[2–7,104,105]
Bingerville	Ivory Coast	16,345–14,976 calBP	–	Guineo-Congolian forest	Undetermined	Quartz (exclusively)	End-scrapers, very small burins, retouched edges, a side-scraper	Freehand direct percussion	Flakes essentially	–	Small assemblage (n = 36)	[106–108]
Toumboura I	Senegal, Falémé valley	–	16 ± 1 ka	Post-LGM/ Pre-AHP open savanna	Local, secondary position	Chert (dominant), quartz, greywacke	Small segments, two backed bladelets	Freehand direct percussion	Flakes, bladelets and blades	Opportunistic approach (unidirectional, bidirectional, or orthogonal), short sequences	Very similar to Ravin de Sansandé	[22]
Iho Eleru	Nigeria	13,452–12,756 calBP	–	Guineo-Congolian forest	Local	Quartz (dominant), chalcedony	Segments, triangles, backed pieces, burins, scrapers	Freehand direct percussion	Flakes essentially, a few bladelets	Low standardization; discoidal and polyhedral cores	Human, faunal and archeobotanical remains	[109–114]
Ravin de Sansandé	Senegal, Falémé valley	–	13–12 ± 1 ka	Post-LGM/ Pre-AHP open savanna	Local, secondary position	Chert (dominant), quartz, greywacke	Small segments, three scrapers	Freehand direct percussion	Flakes, bladelets and blades	Opportunistic approach (unidirectional, bidirectional, or orthogonal), short sequences	Very similar to Toumboura I	[22]
Fatandi V	Senegal, Falémé valley	–	12.8–10.3 ka	Post-LGM/ Pre-AHP open savanna	Local, primary position	Chert (exclusively)	Large segments, retouched edges	Freehand direct percussion	Flakes and bladelets. Standardized blanks (among which rectilinear ones)	Unidirectional knapping, short sequences	Knapping workshop; few retouched artifacts	[21]
Damatoumou	Mali, Ounjougou	9471–9008 calBP	–	Post-LGM/ Pre-AHP open savanna	Local	Quartz (dominant)	Drills, retouched flakes, backed points, notches, denticulates, a scraper	Freehand direct percussion; bipolar percussion on anvil	Standardized blanks (rectilinear flakes)	–	Contemporary with the nearby Neolithic site Ravin du Hibou, but did not yield any neolithic artifacts (ceramics, grinding material, etc.)	[11,12,115–118]
					Local	Sandstone	Massive *rabots*	Freehand direct percussion	Large flakes and retouched natural slabs	–		
**Ravin Blanc X**	**Senegal, Falémé valley**	**9397–8988 calBP**	**–**	**Post-LGM/ Pre-AHP open savanna**	**Local, primary position**	**Quartz (almost exclusively)**	**Backed pieces, notches, micro-scraper, retouched edges**	**Freehand direct percussion**	**Standardized blanks (rectilinear flakes, and thin, elongated products)**	**Significant investment in core preparation and maintenance. Minimal retouch. Core exhaustion**	**Knapping workshop, few retouched artifacts; fireplace**	**This article**

#### The LSA of the Falémé valley.

Until now, three LSA sites had been identified in the Falémé valley: Toumboura I (dated to 16 ± 1 ka, located less than 4 km from RBX-1), Ravin de Sansandé (dated between 13 and 12 ± 1 ka, located less than 8 km from RBX-1), and Fatandi V (dated between 12.8 and 10.3 ka, located about 17 km from RBX-1) (Fig 1B) [21,22].

##### The end of the Pleistocene in the Falémé valley: Toumboura I and Ravin de Sansandé

The sites of Toumboura I and Ravin de Sansandé yielded highly similar lithic industries, characterized in particular by the production of geometric microliths, primarily segments [22]. These industries were exclusively produced using local materials, mainly chert, but also quartz and greywacke. Outcrops and veins of these materials are known within a 15 km radius of these sites [119], with greywacke being even more readily available, as it constitutes the predominant bedrock of the area. However, the presence of alluvial neocortical surfaces in the lithic assemblages suggests that raw materials were collected in secondary positions, likely as river pebbles. This choice may have been motivated by the proximity of the watercourse, in contrast to outcrops sometimes located several kilometers away: the present-day bed of the Falémé River is only 130 m from Toumboura I and 400 m from Ravin de Sansandé. The chert, quartz, and greywacke pebbles collected were used at both sites to produce blades, bladelets, and flakes, following simple and opportunistic reduction methods, sometimes unidirectional, bidirectional, or orthogonal [22]. The segments discovered at these sites were made on both bladelets and flakes, suggesting that more effort was invested in the retouching phase rather than in producing standardized blanks from the outset.

The RBX-1 site is significantly farther from the current riverbed, situated 1.8 km away. Here, the site’s occupants chose to work (almost) exclusively with quartz, whose natural surfaces indicate that it was not collected from alluvial contexts. The homogeneity and apparent high quality of the quartz in the assemblage may explain why raw material was sourced directly from primary deposits, with a deliberate selection of blocks based on these criteria. Such selection would be more difficult in alluvial contexts, where quartz pebbles originate from multiple veins and vary greatly in quality.

Regarding reduction strategies, the comparison of assemblages from the three sites highlights a fundamental difference in the preparation and maintenance of cores. Although some debordant products are present in the assemblages from Toumboura I and Ravin de Sansandé, the core analysis indicates short reduction sequences, with small pebbles quickly abandoned after only a few removals [22]. At RBX-1, however, the exploited quartz blocks were used to produce standardized blanks, to the extent that retouching was generally minimal. In this context, cores were exploited to exhaustion: recurrent accidents on exploitation surfaces, exhaustion of convexities with insufficient remaining material to allow for further maintenance, etc. These complex reduction strategies may have influenced the decision to select medium-sized quartz blocks from vein outcrops rather than small river pebbles. The angular morphology of these blocks required minimal preparation for serial production of standardized blanks, which smaller pebbles would not have allowed.

Keeping in mind that RBX-1 represents a knapping workshop, meaning that many finished products were likely transported away from the site for use, a typological difference is also observed. In addition to segments, the retouched toolkits from the two Pleistocene sites include two backed bladelets at Toumboura I and three scrapers at Ravin de Sansandé. No segments were found at RBX-1, but two backed pieces and a micro-scraper are present in the assemblage. While the scrapers from Ravin de Sansandé are described as microlithic (25 mm long, 20 mm wide, 10 mm thick), the micro-scraper from RBX-1 is even smaller (12 mm long, 15 mm wide, and 4 mm thick). The retouch shaping its front edge appears more marginal compared to the scrapers from Ravin de Sansandé [22]. The two backed bladelets from Toumboura I are described as only marginally retouched, with semi-abrupt modifications that do not alter the distal or proximal terminations [22]. The blanks selected at RBX-1 for backing – namely, a large circular flake and a blade – make direct comparisons difficult given the small number of artifacts. As for the most distinctive retouched pieces in the RBX-1 assemblage, notches, and retouched edges, they are completely absent from the LSA Pleistocene assemblages of the Falémé valley.

Once again, given that RBX-1 is a knapping refuse area, it is expected that only a small number of modified products would be found. Furthermore, the types of blanks sought by the knappers at RBX-1 (rectilinear and parallel products, products with an oblique distal termination and a natural asymmetric point) would have been well-suited for the manufacture of segments. Their absence in the assemblage does not categorically rule out the possibility that such pieces were produced on-site and then transported elsewhere, or that raw blanks were selected and taken away to be retouched elsewhere. However, no artifacts that could support this hypothesis (e.g., segment preforms, broken pieces in the process of being retouched) were found at the site.

Aside from its microlithic nature and a few typological convergences, the RBX-1 site appears quite different from the Pleistocene sites of Toumboura I and Ravin de Sansandé. A significant contrast is observed in the strategies implemented at the three sites. In one case, the proximity of raw material sources seems to have been the most important factor, leading to expedient and opportunistic production of diverse blanks, which were then heavily modified through retouching. In the other case, there was an exclusive selection of high-quality, homogeneous quartz blocks from primary outcrops, with the goal of producing standardized and minimally retouched blanks.

##### The Pleistocene-Holocene transition and Early Holocene in the Falémé valley: Fatandi V

The site of Fatandi V, in the Falémé valley, has undergone a series of OSL datings within its stratigraphic sequence [21]. The dates obtained for the archaeological level range between 14.3 and 10.3 ka. A sample taken 10 cm below this level provided a date of 12.8 ka, allowing the chronological range to be refined to 12.8–10.3 ka with a 95% confidence interval, or 11.9–11.2 ka with a 68% confidence interval. Considering these dates, the occupation of Fatandi V falls within the transition between the Pleistocene and the Holocene, or even into the Early Holocene. More recent than the sites of Toumboura I and Ravin de Sansandé, it yielded a noticeably different LSA industry.

The only raw material exploited at Fatandi V is chert. Previously referred to at the time of the site’s publication as “blue-green jaspoid greywacke,” a petrological re-examination of this rock confirms that it is, in fact, chert [20,28]. The natural surfaces observed in the assemblage suggest that it was collected in primary position, in the form of slabs. The nearest identified chert outcrop in the area is located 2.4 km from the site [119]; additional outcrops farther south, near the village of Alinguel, have also been identified during surveys [21]. However, the site itself is only 160 m from the current Falémé riverbed, where pebbles like those collected at Toumboura I and Ravin de Sansandé were likely accessible. Thus, raw material procurement at Fatandi V appears to have followed a specific strategy, where proximity to the resource was not the primary criterion.

A particular focus is drawn to a chert knapping workshop at Fatandi V (North square). The targeted blanks were primarily bladelets and flakes, produced using various methods. Unlike the diverse and opportunistic strategies observed at Toumboura I and Ravin de Sansandé, a significant portion of blanks at Fatandi V were removed in a systematic, unidirectional manner. The “method 2” of bladelet production, aimed at obtaining broad, rectilinear bladelets, is reminiscent of the reduction sequence identified at RBX-1 for producing rectilinear blanks [21]. However, at Fatandi V, cores associated with this method 2 were only subjected to short exploitation sequences and were abandoned after knapping accidents, without prior preparation or maintenance procedures. This difference in treatment between the two sites may be due to the differing raw materials used. While quartz is generally considered lower quality compared to siliceous rocks like chert, the quartz selected at RBX-1 appears more homogeneous and less fractured. In contrast, chert outcrops in the Falémé valley frequently occur as highly fractured layers, significantly complicating knapping activities.

As at RBX-1, very few retouched pieces were identified within this knapping workshop (n = 9/729 total artifacts). Among them is a segment that is larger than those discovered at Toumboura I and Ravin de Sansandé [21]. It measures 35 mm in length, 22 mm in width, and 7 mm in thickness, whereas segments from the other two sites do not exceed 18 mm in length, 10 mm in width, and 6 mm in thickness. Its 22 mm width and the presence of parallel previous scars on its dorsal face suggest it was likely made on what we would classify as a blade. In this regard, the segment is comparable to the backed blade discovered at RBX-1, although the latter exhibits more marginal retouching. The other retouched pieces in the Fatandi V assemblage consist of variably marginally retouched edges on different blank types.

Thus, the assemblage from layer 2 at RBX-1 appears more similar to that of Fatandi V than to the other two LSA sites in the Falémé valley in several respects: 1) Both sites are knapping workshops where a single raw material was exploited, collected in primary position; 2) The reduction strategies aimed at producing standardized blanks, some of which share similar forms between the two sites; 3) Few finished products were discovered, and some of them exhibit typological similarities. Some of these similarities are likely attributable to the function of these sites: as knapping workshops, a low number of retouched products is an expected outcome. Nevertheless, a broader connection between the two sites should be considered.

RBX-1 and Fatandi V are 17 km apart, while Toumboura I and Ravin de Sansandé are located between them (Fig 1B). The differences observed among these four sites cannot be explained by geography alone. Tentatively, and acknowledging the limited number of available sites, we propose that RBX-1 and Fatandi V may represent a distinct phase of LSA occupation in the Falémé valley, broadly associated with the Pleistocene-Holocene transition and the Early Holocene. This phase could potentially reflect shifts in resource acquisition strategies, land-use patterns, and production objectives. However, this remains a working hypothesis that requires further testing.

We recognize that the similarities between RBX-1 and Fatandi V may be partly explained by their shared function as knapping workshops, where a single raw material was exploited and a similar set of production strategies was employed. These similarities could also be circumstantial, rather than signaling a distinct phase of LSA development. Therefore, while the potential for a technological shift at this time is an intriguing avenue for future research, it is important to consider the differences that persist between these two sites. For instance, not all the methods and blank types are shared between them. The variation in raw materials used at the two knapping clusters also complicates interpretations, as each material presents its own technical constraints, potentially leading knappers to adopt distinct reduction strategies. Lastly, as previously noted, the selective removal of retouched pieces post-production could introduce biases in the representation of certain tool categories over others.

As such, we present this hypothesis as a framework for future comparative analysis, rather than a definitive interpretation. A broader regional dataset and further investigation into the spatial and chronological distribution of these types of assemblages will be necessary to test this hypothesis more rigorously.

#### The LSA in West Africa.

In 1981, T. Shaw proposed the existence of two facies within the aceramic LSA complex of West Africa: a microlithic facies in savanna zones and a non-microlithic facies in forested areas [14]. However, it has since been demonstrated that the diversity of the LSA in the region is not so dichotomous. Nonetheless, current knowledge suggests that the LSA appears earlier in the tropical forested regions of northwestern Central Africa than in the rest of West Africa [2–4]. In the West African savanna zones, a fully developed LSA only seems to emerge after the arid maximum of the LGM, in a context of gradual climatic improvement, with the earliest known LSA site at these latitudes being Toumboura I [12,22].

In the tropical forests of Cameroon, the arid episodes and climatic instability characteristic of MIS 2 appear to have had only a minimal impact on local landscapes [120,121]. It is in this region that the oldest known LSA sites in West Africa are found, namely Shum Laka and Njuinye, where LSA occupations began during MIS 3 and continued into the Early Holocene [2–7,104,105].

The site of Shum Laka in Cameroon is characterized by a rich stratigraphic sequence, with four layers containing LSA-associated remains. Dated between 38,406 and 6,081 calBP, the lithic material from these layers remains remarkably homogeneous over time, showing no major technological changes [7]. The industry is microlithic, primarily made on local quartz, with a lesser presence of basalt, tuff, chert, and obsidian. Most of the assemblage consists of blank fragments and angular waste; cores and retouched products are relatively rare considering the total assemblage, which comprises over 50,000 artifacts. The toolkit includes segments, backed pieces, scrapers, and borers, almost exclusively made on quartz. Although a few bladelet cores have been identified, knapping seems to have been oriented toward flake production via direct percussion; only one quartz core exhibits traces of bipolar percussion on anvil, and some suggest discoidal exploitation strategies [7]. From 6,081–5,896 calBP (7,140 ± 50 BP, UtC-8026), ceramics first appear at the site. The lithic industry also changes from this level onward, with the quartz microlithic industries giving way to larger industries on basalt and tuff, featuring large bifacial pieces and polished tools [104,105].

Near Shum Laka, the site of Njuinye has also yielded an early LSA occupation [4,5]. Layer 3 has been dated using two charcoal samples to between 39,181 and 19,068 calBP (34,700 ± 560 BP and 17,800 ± 180 BP). Two phases have been identified, differing only in the raw materials used: the lower phase exclusively showed quartz pebbles collected near the site, whereas the upper phase primarily relied on quartz but also incorporated about ten other rock types, including obsidian, chalcedony, and quartzite. From a technological perspective, these two phases are extremely similar. They are characterized by a low degree of standardization in knapping methods, cores reduced using freehand direct percussion, and an abundance of knapping debris and unmodified products compared to a relatively small number of retouched tools. The toolkit mainly consists of scrapers, drills, notches, and a few geometric microliths.

Researchers working on these two sites have hypothesized that the environmental and climatic stability of the forested setting surrounding Shum Laka and Njuinye may have contributed to the long-term stability of LSA technological behaviors observed in this sequence [6,7,120–122].

Typical Guineo-Congolian forests form patches extending along the Gulf of Guinea coast, where a few LSA sites have been identified and excavated (Fig 1A). At the site of Bingerville (also known as Bingerville Highway) in Ivory Coast, a small assemblage of 36 microlithic quartz artifacts—initially classified as “Epipaleolithic”—was discovered and dated to 16,345–14,976 calBP (13,050 ± 230 BP, Gif-5626) [106–108]. This assemblage includes end-scrapers, very small burins, a side-scraper, and retouched flakes.

In Nigeria, the site of Iho Eleru (formerly Iwo Eleru) is well known for yielding the only currently known Pleistocene human remains from West Africa [109–112]. Ceramics, faunal remains, archaeobotanical materials (charcoal and carbonized seeds), and shells have also been discovered [113,114], along with an exceptionally large quantity of lithic material (over 360,000 artifacts). The stratigraphically complex sequence at Iho Eleru has been divided, based on studied material and multiple radiocarbon dates, into two phases: phase A, an aceramic phase beginning around 13,452–12,756 calBP (11,200 ± 200 BP, I-1753), and phase B, a ceramic-bearing phase beginning around 8,007–7,685 calBP (7,030 ± 85 BP, Hv-1509). Phase A yielded an assemblage dominated by geometric microliths, about 80% of which are segments, along with triangles and backed pieces. Various types of burins and scrapers complete the toolkit. The cores associated with this phase have been described as mainly discoidal and polyhedral [109]. The raw materials used were predominantly quartz, with chalcedony in smaller amounts. However, precise comparisons with other sites remain difficult, as the lithic study of Iho Eleru was primarily typological. The authors refer to “flaked quartz” and “flaked chalcedony” without further details on the types of blanks being produced. Based on illustrations of the artifacts, some cores exhibit relatively elongated negatives, suggesting sporadic bladelet production, though this does not appear to have been a widespread practice [109]. Overall, knapping activities at the site seem to have been primarily oriented toward flake production, from which finished tools were then made. The illustrations of the microliths confirm this observation: very few rectilinear and parallel scars and ridges are visible on the dorsal faces of the artifacts. Their general dimensions and morphology appear quite variable, possibly reflecting an absence of standardization in tool production.

The LSA sites discovered in Guineo-Congolian forest contexts span a vast timeframe and geographical area. They share a common emphasis on microlithism and the predominant use of quartz in tool production. However, they differ typologically, with toolkits varying from site to site—potentially reflecting functional differences—and in the methods and techniques employed. When knapping techniques are described, freehand direct percussion appears to have been the dominant approach, while bipolar percussion on anvil is only sporadically present. The collected blocks and/or pebbles were sometimes exploited unidirectionally, while other times they followed discoidal or polyhedral reduction strategies, most often producing unstandardized flakes. These flakes served as blanks to produce geometric microliths but also scrapers, drills, notches, burins, and various other retouched flakes.

At most of the mentioned sites, the limited focus on blank standardization and the prominence of segment production resemble the assemblages of Toumboura I and Ravin de Sansandé in the Falémé valley. However, they contrast sharply with the assemblages from RBX-1 and Fatandi V, where the primary objective appears to have been the production of standardized blanks, which were only marginally retouched to create a less diverse range of tools than those described at the various forest sites.

#### The question of the persistence of MSA behaviors.

Recent publications suggest that MSA behaviors persisted until the Early Holocene. This idea is currently based on two main sites: Ndiayène Pendao and Saxomununya in Senegal [123,124]. The occupation at Ndiayène Pendao is characterized by the use of Levallois knapping methods, employed to produce points and preferential flakes, as well as blade production. The toolset includes denticulates, core axes, and bifacial thinning flakes. This occupation has been dated by OSL to 11.6 ± 0.51 ka (SHFD15010). At Saxomununya, most of the cores show Levallois reduction methods, with some discoidal techniques also present. The resulting products were used to manufacture denticulates, end-scrapers, side-scrapers, notches, and retouched Levallois flakes. This occupation has yielded a date of 11.1 ± 0.58 ka.

However, these two sites come from contexts that raise several questions. Only two OSL dates have been obtained from Ndiayène Pendao in relation to the archaeological material, and they are stratigraphically inverted; the more recent date was retained, while the other was 22.7 ± 1.2 ka (SHFD15011). Obtaining new dates from this sequence would help clarify the site’s sedimentary dynamics. Most of the material from Saxomununya comes from surface collection, while only five artifacts were found in stratigraphy, within a layer of coarse alluvium linked to significant fluvial activity. Typo-technological similarities between these five artifacts and those found on the surface led the authors to consider them as part of a homogeneous assemblage. However, although the artifacts recovered in stratigraphy exhibit a fresh surface condition, this type of discovery context calls for caution in interpreting the obtained OSL date and the in situ nature of the material.

In any case, the emergence of the LSA during MIS 3 in the tropical forests of Cameroon is contemporaneous with “non-LSA” sites in the Sahelo-Sudanian zones of West Africa [10,65,94]. The site of Laminia, dated to MIS 2 (22.0 ± 0.85 ka and 20.8 ± 0.83 ka), has yielded Levallois artifacts considered “typical” of the MSA [124]. In the lower Falémé valley, a different trajectory is observed. Here, the most recent site with formally MSA characteristics, Toumboura III, is dated to the end of MIS 3, around 37 ± 3 ka [65]. During MIS 2, the sites of Toumboura II (29 ± 2 ka) and Ravin des Guêpiers (18 ± 1 ka) indicate a continued occupation of the valley throughout this period [94,125]. However, their assemblages, primarily characterized by what appears to be an opportunistic reduction strategy and a limited toolset composed of notches, denticulates, and scrapers, raise questions regarding their techno-cultural attribution and the broader definitions of the MSA and the LSA [126]. Only afterward do the LSA sites of Toumboura I and Ravin de Sansandé appear, dated to the late MIS 2, and described earlier.

If we accept the chronology proposed for Ndiayène Pendao and Saxomununya, the existence of MSA sites dated to the Early Holocene, in regions where LSA sites were already established, would suggest the cohabitation of groups with different traditions and technical knowledge. At the same time, in other parts of Africa, the replacement of MSA behaviors by LSA traditions had already occurred long before [127–129]. The discovery of solid new contexts confirming the persistence of these MSA behaviors into the Holocene would have decisive implications for our understanding of these groups and their territorial occupation strategies in the highly dynamic environment of the Pleistocene-Holocene transition. In any case, the knapping methods and production objectives identified at RBX-1 are quite distinct from those described for West African MSA sites and instead align with the well-documented microlithization process characteristic of the region’s LSA.

#### The earliest ceramic industries of West Africa.

The LSA industry at the RBX-1 site is chronologically later than the earliest ceramics discovered in West Africa. Therefore, the coexistence of the earliest ceramics with the persistence of behaviors that appear to belong to an aceramic LSA raises several questions and has multiple implications, making it relevant to discuss the West African ceramic sites dated to the Early Holocene.

In the Yamé valley in Mali, within the Ounjougou archaeological complex, the return of more favorable climatic conditions during the Early Holocene coincides with the end of the sedimentary and/or occupational hiatus of the LGM [8]. Phytolith studies conducted on several excavated sites suggest a landscape of open grassland savanna, where trees were rare and limited to gallery forests along rivers and ponds [93,130]. The two main sites documenting the emergence of ceramics, Ravin de la Mouche and Ravin du Hibou, are located on the right bank of the Yamé, less than 50 meters apart, and their stratigraphies have been interpreted together [12,13,117,118].

At Ravin de la Mouche, layer HA1 has yielded a rich lithic industry, including bifacially retouched armatures with marginal or covering retouch, sometimes shaped by pressure flaking, and devoid of geometric microliths [12,13,117,118]. Three ceramic sherds were discovered at the base of this level (HA1A), with a *terminus ante quem* dating the overlying layer HA2–11,395–10,581 calBP (9510 ± 70 BP, ETH-31279; 9785 ± 70 BP, ETH-28746).

At Ravin du Hibou, an assemblage containing ceramic sherds and grinding equipment was uncovered within unit HA4 and dated by five charcoal samples and two OSL dates to approximately 9913–8766 calBP, confirming the antiquity of ceramics at Ounjougou [12,13] (Radiocarbon dates: 8700 ± 75 BP (ETH-20214), 8210 ± 60 BP (Ly-6804), 8115 ± 50 BP (Ly-9339), 8085 ± 50 BP (Ly-9335) and 8080 ± 55 BP (Ly-9334). OSL dates: 11 ± 1,1 ka (00/5/4) and 9,8 ± 1 ka (01/5/4)). This assemblage also includes lithic material, mostly quartz, with a significant proportion of unmodified cortical elements and angular waste, suggesting that at least part of the knapping took place on-site [117]. Evidence of bipolar percussion on anvil has been observed on some artifacts; however, the small number of identified cores (n = 6) limits our understanding of the reduction sequences. Similarly, retouched pieces are scarce (n = 23) and mainly consist of various retouched flakes (n = 14), two scrapers, and fragments of bifacially retouched tools (n = 7) [12,117].

Located on a terrace on the left bank of the Yamé, about 200 meters from Ravin du Hibou, the site of Damatoumou has yielded two lithic knapping workshops situated 20 meters apart within the same stratigraphic level, dated between 9471 and 9008 calBP (8300 ± 70 BP, ETH-20213; 8210 ± 70 BP, ETH-18652). Although contemporary with Ravin du Hibou, no ceramic or grinding material has been discovered there, and differences in lithic material are also observed between these two sites.

The first of the two knapping workshops, Damatoumou 1 (previously named “Ounjougou-5”) [11,12,115–118], covering approximately 560 m², features a dominant quartz pebble industry (about 80% of the assemblage) alongside a component of large sandstone artifacts (about 20% of the corpus). Only one sandstone core has been found, but the study of the 195 quartz cores indicates unidirectional knapping, with over a quarter exhibiting traces of bipolar percussion on anvil. Many of the quartz flakes are short, wide, and rectilinear, with rectilinear flake scars on their dorsal face. Furthermore, 38% of these flakes are hinged, and 80% of the cores display hinge scars on their exploitation surface, though it remains unclear whether this was an unintended limitation or an indication of a lack of interest in producing elongated blanks [115]. Despite differences in collected raw material size and knapping methods, similarities with the contemporaneous RBX-1 assemblage emerge. In terms of tools, the retouched quartz pieces include drills, retouched flakes, backed points, notches, denticulates, and a scraper. Sandstone tools, on the other hand, consist exclusively of “massive *rabots*” [12]. These retouched products confirm a knapping economy aimed at producing a diverse and complementary toolset depending on the raw materials used.

The second knapping workshop, Damatoumou 2, contains a lithic artifact concentration over approximately 2 m², primarily consisting of quartz, with smaller amounts of sandstone and flint [12,118]. Here again, unidirectional knapping dominates, while the use of bipolar percussion on anvil is less present compared to Damatoumou 1. The fundamental difference between the two assemblages lies in the retouched material: at Damatoumou 2, there is no large sandstone macro-tool industry, and only a microlithic quartz component is present among the retouched material. A few retouched flakes, drills, backed points, and notches are accompanied by geometric microliths, including segments, crescents (i.e., segments with additional retouch on the opposite edge), trapezoids, and a triangle; a bifacially retouched flake completes this assemblage.

Thus, despite their contemporaneity and geographical proximity, the two sites appear quite different: on one hand, Ravin du Hibou features bifacial tools, ceramics, and grinding equipment; on the other, Damatoumou is characterized by the predominance of geometric microliths and, to a lesser extent, sandstone macro-tools. However, some common points stand out: predominant use of quartz, primarily unidirectional knapping, and significant use of bipolar percussion on anvil [117]. Based on these technical similarities, the authors propose interpreting these sites as linked to the same human groups but serving distinct functions. Damatoumou would therefore be a knapping site, specialized in a particular lithic production, while Ravin du Hibou would represent a “habitation” site, where more diverse activities occurred [12,117]. Similarly, the layer 2 assemblage at RBX-1, representing a short-term knapping site, could be the result of a specific activity carried out by a group that mastered ceramic technology and used it elsewhere.

In the Falémé valley, the only Neolithic site found in stratigraphy is layer 4 of RBX-1, dated to 3173–2998 calBP. This layer follows a 6000-year sedimentary hiatus after layer 2 of RBX-1, which represents the most recent LSA occupation in the valley [131]. However, this does not exclude the possibility that older ceramic sites may have existed in the valley. This is suggested by the discovery at the site of Fatandi V of a few ceramic sherds tempered with a white mineral and bearing an unidentifiable decoration, which were found stratified beneath the upper glacis and associated with a radiocarbon date of 5029–4845 calBP (4346 ± 33 BP, ETH-55081) [21,27]. This is also the case of a rim fragment from a vessel with an everted lip, tempered with grog and quartz, and decorated with a fine comb-impressed pattern, which was found at Toumboura I in a level dated to 6396–6298 calBP (5556 ± 26 BP, ETH-87708) [22].

Nevertheless, based on current knowledge, there is no evidence to suggest any contemporaneity between the LSA and the Neolithic in the area. The industries from the two layers of RBX-1 differ significantly in many respects, including the materials used and the intended purpose of the knapping activities. The 6000-year hiatus between them currently prevents us from formulating hypotheses regarding the emergence of Neolithic behaviors in the Falémé valley, such as ceramics, polishing, and grinding tools.

## Conclusion

Dated to the Early Holocene, the assemblage from layer 2 of Ravin Blanc X-1 represents the most recent Later Stone Age occupation identified to date in the Falémé valley, eastern Senegal. Preceding the only well-characterized Neolithic occupation in the valley after a nearly 6000-year hiatus, it fills a gap in the region’s long prehistoric sequence and contributes to our still incomplete understanding of the West African LSA.

This occupation is characterized by a fireplace associated with a quartz knapping workshop. The quartz workshop consists of a dense concentration of almost 1,200 artifacts. 80% of these artifacts are knapping waste, attesting to the function of this concentration as a knapping workshop. Their presence within the workshop, despite their very small dimensions, confirms what grain size and anthracological analyses have also suggested, i.e., that the site was only slightly impacted by taphonomic phenomena. In a region where stratified sites are rare, the exceptional preservation of this site has provided a unique opportunity for extensive multidisciplinary studies, offering the most detailed possible view of the techno-cultural traditions of its inhabitants and the paleoenvironmental context in which they lived. The presence of the fireplace itself—an extremely rare, if not unique, vestige of this kind for the Paleolithic in West Africa—also attests to the extremely limited impact of post-depositional processes.

A comparison of this assemblage with those currently known and described for the LSA and Early Holocene in West Africa leads to several observations:

1] Since the emergence of the LSA in West Africa during MIS 3, there has been a general trend toward the microlithization of industries, aiming to produce a diversified set of retouched tools in which geometric armatures are predominant. The exploited raw materials are systematically local, with quartz being preferentially selected at most sites. The collected blocks are worked using varied methods, most often direct freehand percussion rather than bipolar percussion on an anvil. Most of the well-documented and stratified LSA sites in the region exhibit several, if not all, of these characteristics. This general trend is reminiscent of what K. C. MacDonald previously proposed as the West African Microlithic Technocomplex [16].
2] Despite general similarities, the West African LSA is far from uniform. While geometric microliths appear to be the primary objective of knapping activities, they are not present at all sites or in the same proportions. The rest of the toolkit is highly variable, including end-scrapers, side-scrapers, borers, notches, depending on the site. The methods and techniques used for debitage are also extremely diverse, reflecting different objectives and even different conceptions of knapping. Approaches to volume management vary, leading to the production of different blanks, and the application (or absence) of retouch differs from one site to another.
3] While distinctions between MSA, LSA, and Neolithic assemblages are generally clear, the processes and timing of transitions between these phases remain difficult to trace. The MSA sites described in West Africa exhibit characteristics very distinct from those of LSA sites: larger tool dimensions, bifacial shaping, the use of Levallois and discoidal methods, a greater reliance on bipolar-on-anvil percussion, etc. The same applies to certain Neolithic sites, whose lithic assemblages differ fundamentally from those of LSA sites. In addition to the adoption of ceramics and macrolithic grinding tools, these Neolithic sites show an abandonment of microlithization in favor of a toolkit that includes bifacial points, among other elements. In areas where similarities between MSA, LSA, and Neolithic assemblages appear to exist, sites are unfortunately too scarce or separated by a hiatus of several thousand years (e.g., the MSA-LSA transition at Ounjougou in Mali, the LSA-Neolithic transition in the Falémé valley in Senegal), making it difficult to interpret typo-technological transition processes between these phases.
4] Possible phases of contemporaneity—between MSA and LSA at the end of the Pleistocene, and between LSA and the Neolithic at the beginning of the Holocene—further highlight these differences, suggesting distinct techno-cultural trajectories among human groups. The evidence points to real ruptures between these different complexes, reinforcing the sense of coherence within a “West African microlithic LSA complex,” within which regional and/or chronological facies certainly existed.

In our study, we observe that the assemblages most like RBX-1 from a typo-technological perspective are those of the Sahelo-Sudanian sites of Fatandi V and Damatoumou. In contrast, LSA and/or contemporary sites from the Guineo-Congolian forest zone appear to reflect knapping concepts very different from those described at these three Sahelo-Sudanian sites. Could the similarities among these three sites indicate connections between human groups that inhabited the Falémé valley in Senegal and the Yamé valley in Mali? More broadly, could these commonalities reflect the existence of a Sahelo-Sudanian facies in the Early Holocene of West Africa, representing a local expression of transition processes between a Late LSA and an Early Neolithic?

The discovery of this new site within such a rich sequence in the lower Falémé valley provides an unprecedented resolution for this period in West Africa. This sequence offers the opportunity for inter-site comparisons in a context where human groups occupied the same territory and had access to the same resources. However, the uneven nature of currently available data makes it difficult to fully understand the dynamics underlying this diversity within the LSA on a regional scale as vast as West Africa. A denser network of sites is needed to confirm, refute, or refine the models and hypotheses proposed thus far.

## Supporting information

S1 Table Detailed typo-technological study of lithic material from layer 2 of RBX-1. (XLSX)

S1 File Inclusivity in global research questionnaire. (DOCX)
